# Genome-wide and expression pattern analysis of JAZ family involved in stress responses and postharvest processing treatments in *Camellia sinensis*

**DOI:** 10.1038/s41598-020-59675-z

**Published:** 2020-02-17

**Authors:** Yucheng Zheng, Xuejin Chen, Pengjie Wang, Yun Sun, Chuan Yue, Naixing Ye

**Affiliations:** College of Horticulture, Key Laboratory of Tea Science, Fujian Agriculture and Forestry University, Fuzhou, Fujian 350002 China

**Keywords:** Gene expression, Gene expression, Jasmonic acid, Jasmonic acid

## Abstract

The *JASMONATE-ZIM DOMAIN* (*JAZ*) family genes are key repressors in the jasmonic acid signal transduction pathway. Recently, the JAZ gene family has been systematically characterized in many plants. However, this gene family has not been explored in the tea plant. In this study, 13 *CsJAZ* genes were identified in the tea plant genome. Phylogenetic analysis showed that the JAZ proteins from tea and other plants clustered into 11 sub-groups. The CsJAZ gene transcriptional regulatory network predictive and expression pattern analyses suggest that these genes play vital roles in abiotic stress responses, phytohormone crosstalk and growth and development of the tea plant. In addition, the *CsJAZ* gene expression profiles were associated with tea postharvest processing. Our work provides a comprehensive understanding of the *CsJAZ* family and will help elucidate their contributions to tea quality during tea postharvest processing.

## Introduction

Higher plants face a large number of severe challenges during their life cycles, including insect bites, pathogen infection, heavy metal stress, and water scarcity. However, as sessile organisms, plants have evolved sophisticated mechanisms to resist these problems and clever strategies to thrive in their ever-changing natural environments^1,2^. For plants, phytohormones are the most effective and fastest weapon in response to environmental stress. For example, jasmonic acid (JA) is widely known to play an important role in various biological processes in plants, including defense against herbivorous insect attack, flower initiation and plant morphogenesis^3–5^.

There are at least two jasmonate synthesis pathways that exist in plants, namely octadecane pathway and hexadecanoid pathway, which begins with the release of α-linolenic acid (18:3n-3) and hexadecatrienoic acid (16:3n-3), respectively^6^. The unsaturated fatty acids are catalyzed by a series of enzymes then generates 12-oxo-10,15 (Z)-phytodienoic acid (OPDA), in the chloroplast^7^. Finally, JA is formed through OPDA reductase 3 (OPR3)-mediated reduction reaction and three rounds of β-oxidation^6^. Interestingly, an alternative pathway for JA biosynthesis was discovered. OPDA could enter the β-oxidation pathway to produce a direct precursor of JA and JA-lie in the absence of OPR3^8^. Moreover, the JA signal pathway has been deciphered^9,10^. It has been reported that the JA receptor is a co-receptor complex formed by JAZ protein, COI1 (*CORONATINE INSENSITIVE1*) protein and inositol pentakisphosphate^11^. Previous studies have revealed that the JA content is maintained at a relatively low level in plants under normal conditions, in which the JAZ repressor interacts with MYC2 to inhibit downstream insect-resistant or disease-resistant gene expression^12^. However, large amounts of JA accumulate in plant cells in response to abiotic or biotic stresses and are perceived by COI1^13,14^. Subsequently, JAZ proteins are degraded by COI1-mediated E3 ubiquitination. Then, the transcription activator MYC is relieved, which increases the expression of downstream JA response genes^15,16^. In short, the JAZ repressor plays an important role in the signaling cascades triggered by jasmonates^12,17^.

The JAZ protein family contains the highly conserved TIFY domain (ZIM) with the consensus sequence TIF[F/Y]XG at the N-terminus and the Jas domain (CCT_2) with the consensus sequence SLX_2_FX_2_KRX_2_RX_5_PY at the C-terminus^12^. These two core highly conserved domains play irreplaceable roles in the JA signal transduction pathway. The interactions among *JAZ* and other genes, such as *COI1* and *MYC*, are mediated by the Jas domain^11^. Additionally, the TIFY domain has been found to interact with NINJA, which is a novel interactor with JAZ, and these genes play a joint inhibitory role in the JA signal transduction pathway^18^. Moreover, research has indicated that loss of the Jas domain confers insensitivity to plant resistance to insects^19^.

Recent studies have shown that individual *JAZ* genes perform specific functions in different plant tissues. In *Arabidopsis thaliana*, *JAZ*2 is exclusively expressed in stomatal guard cells and indirectly regulates *ANAC* expression to regulate the stomatal aperture^20^. In *Nicotiana attenuate, NaJAZd* plays an important role in shedding of flower buds^21^. Moreover, JAZs are involved in controlling the crosstalk between the JA signal transduction pathway and other phytohormones in response to stresses^22^. A typical example is the horizontal JAZ-EIN3 interaction, which serves as the bridge between the JA and ET pathways^23^.

Tea is an important cash crop that is known for its distinctive flavor. A recent study showed that treatment of fresh tea leaves with methyl jasmonate could greatly increase the volatile aromatic content of some tea products^24^. Some genes related to the terpenoid backbone biosynthesis pathway were upregulated under exogenous application of MeJA^25^. However, *CsJAZ* genes have not been reported in the tea plant to date. In particular, JA may contribute to the tea aroma, but the effects of postharvest treatments on JA signal transduction during oolong tea processing remain unknown. In this study, we conducted a comprehensive analysis of the architecture and function of the *CsJAZ* gene family, including prediction of phylogenetic relationships, characterization of functional domains, and promoter analysis. Additionally, the expression patterns in response to plant hormone and in eight typical tissues were investigated, and the expression profiles of the *CsJAZ* genes during postharvest rotating treatment of oolong tea were analyzed. Our analysis provided comprehensive insights into the biological functions of the *CsJAZ* family and elucidated the important role of the *CsJAZ* family in oolong tea processing.

## Results

### Identification of the JAZ family in *C. sinensis*

To identify all *CsJAZ* family genes more comprehensively, three typical methods (hmmsearch, Blastp search and annotated JAZ genes in TIPA (tea plant genome database)) were used. Redundant sequences were manually removed. The Jas and TIFY domains were validated by CDD (http://www.ncbi.nlm.nih.gov/Structure/cdd/) and SMART (http://smart.embl-heidelberg.de/). As a result, a total of 13 *CsJAZ* genes were identified. The genomic length of the 13 *CsJAZ* genes ranged from 16862 bp (JAZ3) to 1581 bp (CsJAZ7) with CDS lengths of 1386 bp (CsJAZ3) to 411 bp (CsJAZ7). The ORF amino acid lengths of the CsJAZs ranged from 461 aa to 136 aa with molecular weights of 49.39 kDa (CsJAZ3) to 15.60 kDa (CsJAZ7). The predicted PI of JAZ7 was highest, and those of CsJAZ7, CsJAZ1, CsJAZ8, CsJAZ9, CsJAZ11, CsJAZ12 and CsJAZ13 were similar; all of these proteins were basic. The predicted PIs of CsJAZ2-6 and CsJAZ10 were less than 7.0, indicating that these proteins were acidic. The grand average of hydropathicity (GRAVY) values of all CsJAZ proteins ranged from −0.916 to −0.310, indicating that the 13 CsJAZ proteins were hydrophilic, with GRAVY values less than zero (Table 1).

**Table 1:** Summary information of *CsJAZ* genes in tea plant.

Locus ID	Gene	Genomic(bp)	CDS(bp)	PI	MW(Da)	ORF(aa)	GRAVY
TEA001681.1	*JAZ1*	6873	1233	9.34	42846.63	410	−0.310
TEA002032.1	*JAZ2*	5544	1152	4.76	41644.84	383	−0.795
TEA001414.1	*JAZ3*	16862	1386	5.75	49389.47	461	−0.375
TEA033836.1	*JAZ4*	6893	816	4.57	30518.72	271	−0.601
TEA033832.1	*JAZ5*	7644	1158	4.88	42000.50	385	−0.625
TEA014550.1	*JAZ6*	2655	705	5.00	25201.85	234	−0.550
TEA004474.1	*JAZ7*	1581	411	9.93	15604.98	136	−0.721
TEA032228.1	*JAZ8*	2534	663	9.18	24453.53	220	−0.557
TEA001501.1	*JAZ9*	4372	786	9.17	29266.15	261	−0.666
TEA013465.1	*JAZ10*	4336	912	6.19	33496.78	303	−0.916
TEA001821.1	*JAZ11*	7825	1311	9.58	46207.07	436	−0.343
TEA030190.1	*JAZ12*	2682	885	9.46	31531.45	294	−0.395
TEA027049.1	*JAZ13*	3489	639	9.15	23877.91	212	−0.632

### Phylogenetic and classification analyses of the CsJAZ members

To understand the evolutionary relationships among the CsJAZ proteins, a total of 75 JAZ proteins from 8 representative plants, including a bryophyte (*P. patens*), lycopodiophyte (*S. moellendorffii*), gymnosperm (*P. abies*), monocots (*O. sativa* and *S. bicolor*) and eudicots *(P. trichocarpa*, *A. thaliana*, and *C. sinensis*), were used for the phylogenetic analysis. We used 12 AtJAZ proteins as queries against the algal genomic databases on the JGI website using the Blastp program. The algal genomic databases included *Ostreococcus tauri*, *Chlorella variabilis* NC64A, *Volvox carteri*. No JAZ proteins were found in the algal genomic databases. This result indicates that JAZ genes probably exist only in terrestrial plants.

**Figure 1:**
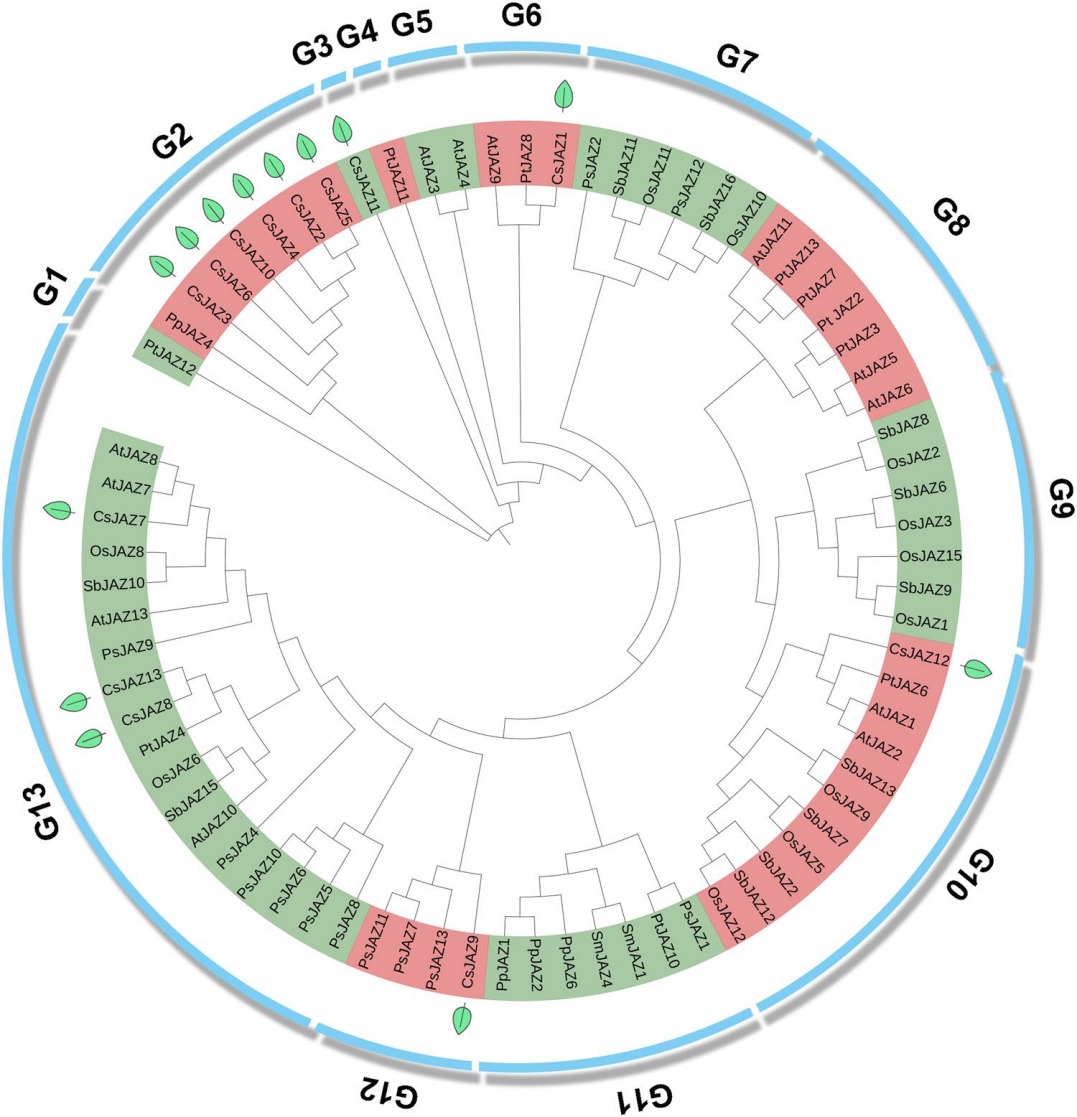
Phylogenetic analysis of the CsJAZ family. The maximum-likelihood method with 1000 bootstrap replications was performed in the MEGA X software. Different colors between adjacent pairs represent different sub-group, and the green leaves represent the 13 *CsJAZ* genes.

The JAZ proteins from the 8 different species were divided naturally into 13 sub-groups (G1-G13) (Fig. 1). Among the 13 sub-groups, the G2 group had the maximal number of CsJAZ proteins, including CsJAZ2-6 and CsJAZ10. The G13 group contained CsJAZ7-8 and CsJAZ13. In addition, the G3, G6, G10 and G12 groups contained the CsJAZ11, CsJAZ1, CsJAZ12 and CsJAZ9 proteins, respectively. Moreover, each CsJAZ protein was clustered with its possible homologs. For example, CsJAZ2-6 and CsJAZ10 were clustered into the G2 group with the bryophyte *Physcomitrella patens*. CsJAZ12 was clustered into the G12 group with the gymnosperm *Picea abies*. CsJAZ7-8, CsJAZ12-13 and CsJAZ1 were clustered with the eudicots *Populus trichocarpa* and *Arabidopsis thaliana*. Therefore, CsJAZ2-6 and CsJAZ10-11 were artificially divided into the ancient group, whereas CsJAZ1, CsJAZ7-9 and CsJAZ12-13 were artificially divided into the modern group. The results may indicate the evolutionary history of the CsJAZ families.

### Structural and conserved motif analyses of the CsJAZ members

**Figure 2:**
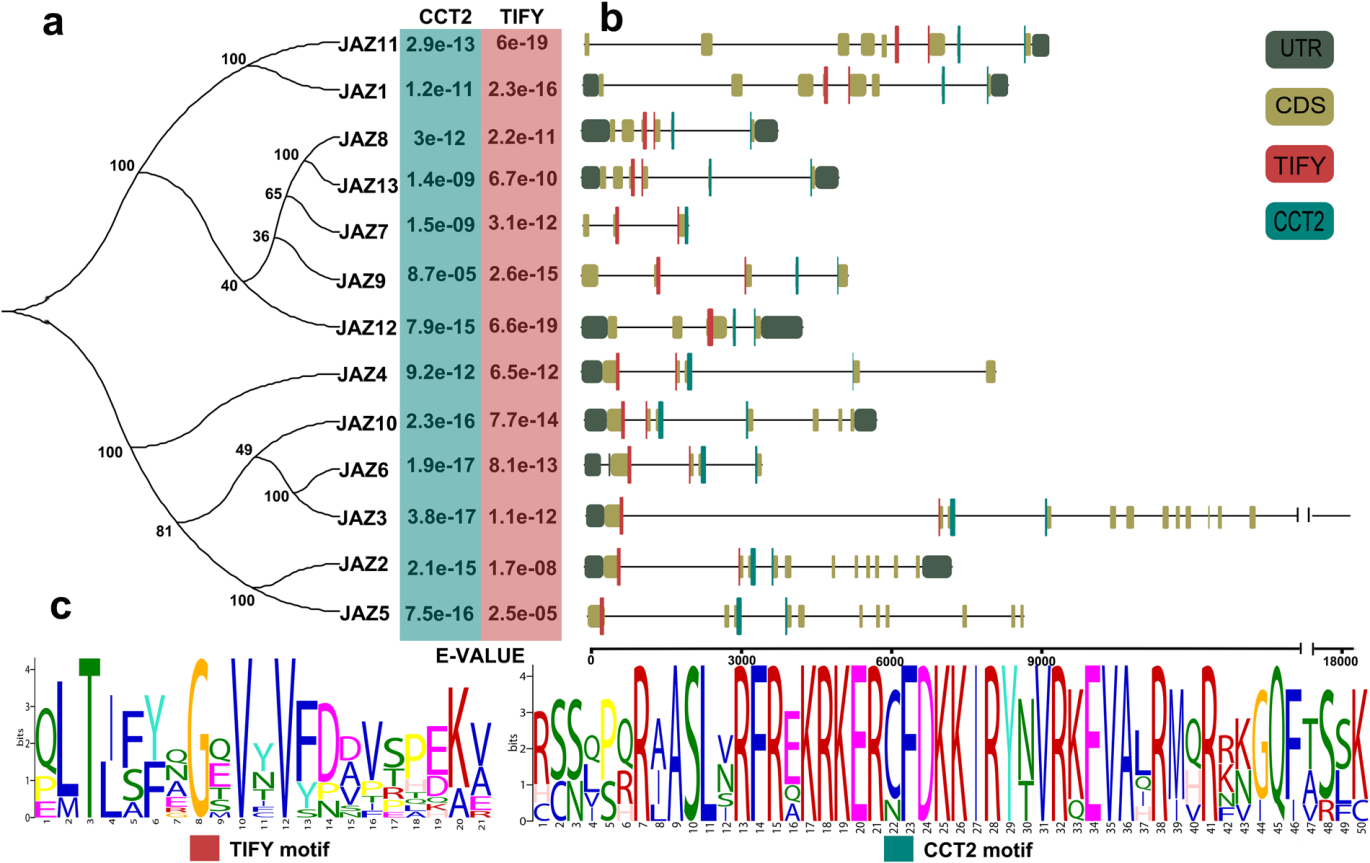
Exon-intron and motif structures of CsJAZ. (**a**) The phylogenetic tree of the CsJAZ members calculated using the neighbor-joining method. (**b**) Domain and exon-intron distribution information was obtained from the tea plant genome database and visualized using the TBtools software. (**c**) The seqLogos of the TIFY and CCT2 motifs.

To better understand the structural diversity among the CsJAZ proteins, the exon-intron structures and a phylogenetic tree were analyzed. The number of exons in the CsJAZ proteins ranged from 3 (CsJAZ7) to 12 (CsJAZ3). In detail, CsJAZ4, CsJAZ6-7, CsJAZ9 and CsJAZ12 contained 3-5 exons, whereas CsJAZ1, CsJAZ8, CsJAZ10-11 and CsJAZ13 contained 5–8 exons. In addition, we found that all of the CsJAZ proteins had the conventional N-terminal TIFY domain and C-terminal CCT2 motif (Fig. 2b). The seqLogos of the TIFY domain and CCT2 motif generated using all CsJAZ protein sequences revealed that the two motifs were well conserved in the tea plant (Fig. 2c). Interestingly, with the exception of the TIFY motif in CsJAZ5 and CsJAZ12, introns were inserted into all of the TIFY and CCT2 motifs in the CsJAZ family.

**Figure 3:**
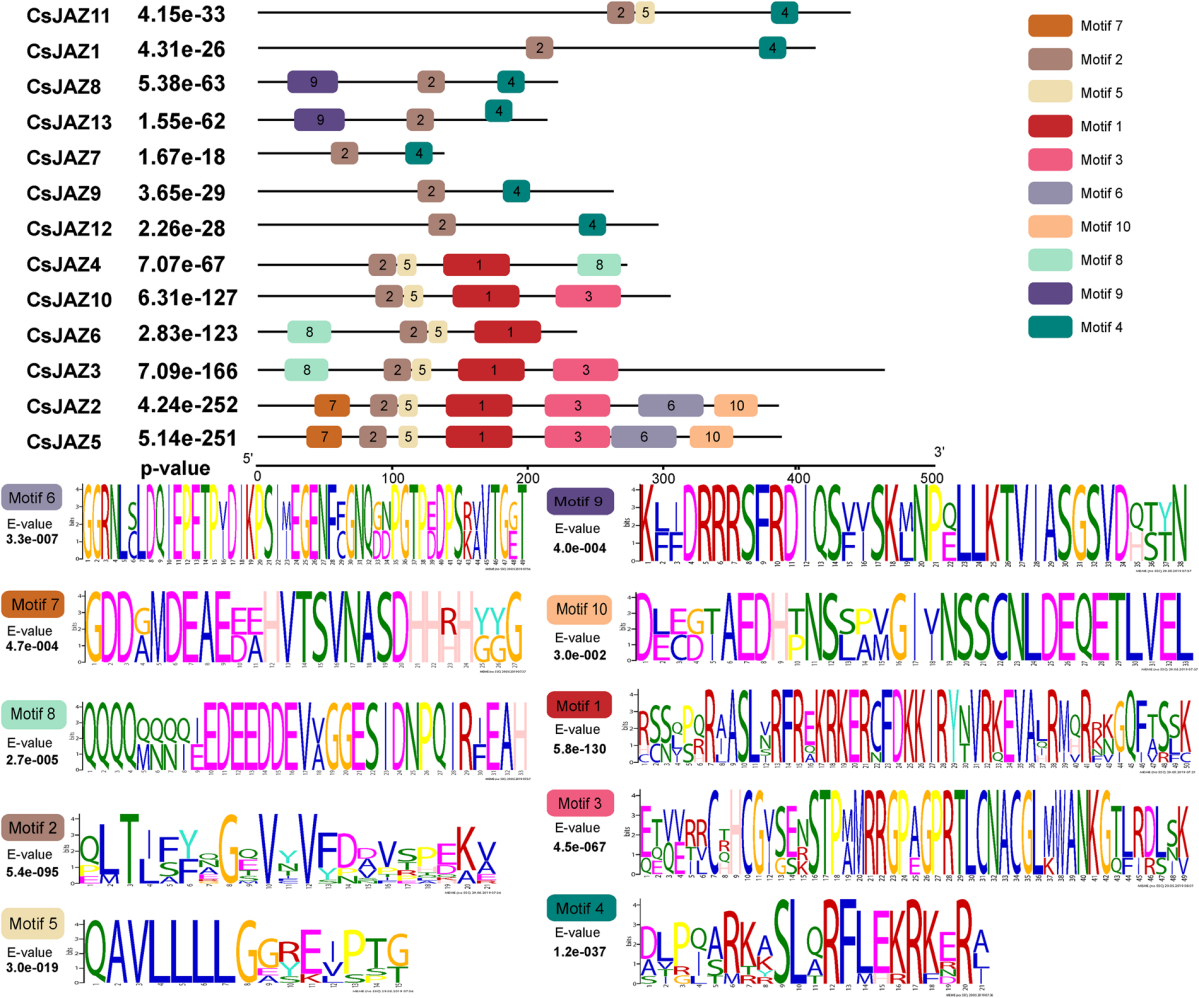
Conserved motif analysis of the CsJAZ proteins in the tea plant. The order of CsJAZ genes are based on the results of the phylogenetic tree. (**a)** Different motifs are shown with various colors. (**b)** Sequence logos of the ten conserved motifs.

Next, the 13 CsJAZ proteins were subjected to conserved motif analysis using the MEME website, and the 10 most conserved motifs were identified (Fig. 3). We found that some specific motifs existed among different CsJAZ family members. For example, motifs 1, 3, 6, 7 and 10 only existed in the ancient group. The results suggest that these motifs have been preserved for a long time.

### *Cis*-Element analysis of the *CsJAZ* genes

**Figure 4:**
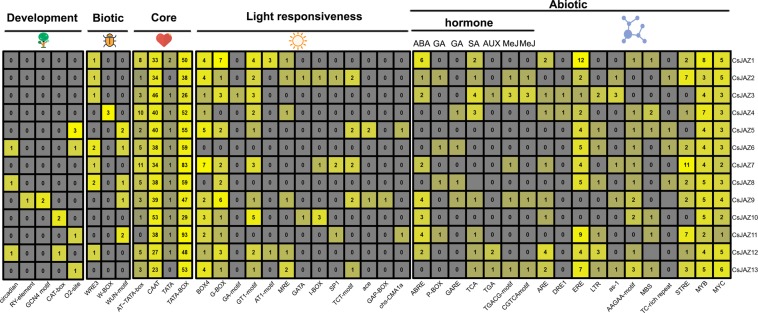
Analysis of cis-elements in the *CsJAZ* genes. Grey indicates the absence of cis-elements upstream of the *CsJAZ* gene. The degree of yellow represents the number of cis-elements upstream of the CsJAZ gene.

Many cis-elements are present in the 1000 bp region upstream of genes and play important roles in the functions of genes involved in plant stress responses and growth. Therefore, we conducted a *cis*-element predection analysis using the 1000 bp region upstream of the *CsJAZ* genes at the PLANTCARE website. According to the function of each *cis*-element, we divided the prediction results into the following groups: plant development, biotic, core element, light responsiveness and abiotic. We found that some abiotic elements, such as MYB, MYC, ERE and STRE, were abundant in the *CsJAZ* genes. Except for *CsJAZ5*, all of the *CsJAZ* genes contained many plant hormone-responsive elements, including ABA (ABRE), GA (P-BOX and GARE), SA (TCA), AUX (TGA) and MeJA (TGACG-motif and CGTCA-motif). These results indicated that the *CsJAZ* family might be involved in the complex hormone regulatory network. A large number of light responsive *cis*-elements, such as BOX 4 and G-BOX, were also found in most of the *CsJAZ* genes. Some elements closely related to plant growth and development were found to be distributed on the CsJAZ promoter. For example, the O2-site, which is a zein metabolism regulatory element, was found only in *CsJAZ5*, *CsJAZ6*, *CsJAZ11* and *CsJAZ13*, whereas the RY-element, which is an element related to plant seed and shoot development, was found only in *CsJAZ10*. Circadian, which is the circadian control element, was found in the promoters of *CsJAZ6*, *CsJAZ8* and *CsJAZ12*. These results suggest that the *CsJAZ* family plays an important role in tea plant growth and development. Meanwhile, some biotic elements, including WRE3 and WUN-motif, were distributed in a small number of *CsJAZ* genes (1–2), and W-BOX was found only in *CsJAZ*4. Additionally, many core promoter elements, such as CCAT and TATA-BOX, were ubiquitously distributed among the *CsJAZ* genes (Fig. 4).

### Identification and annotation of the CsJAZ gene putative transcription factor regulatory network

**Figure 5:**
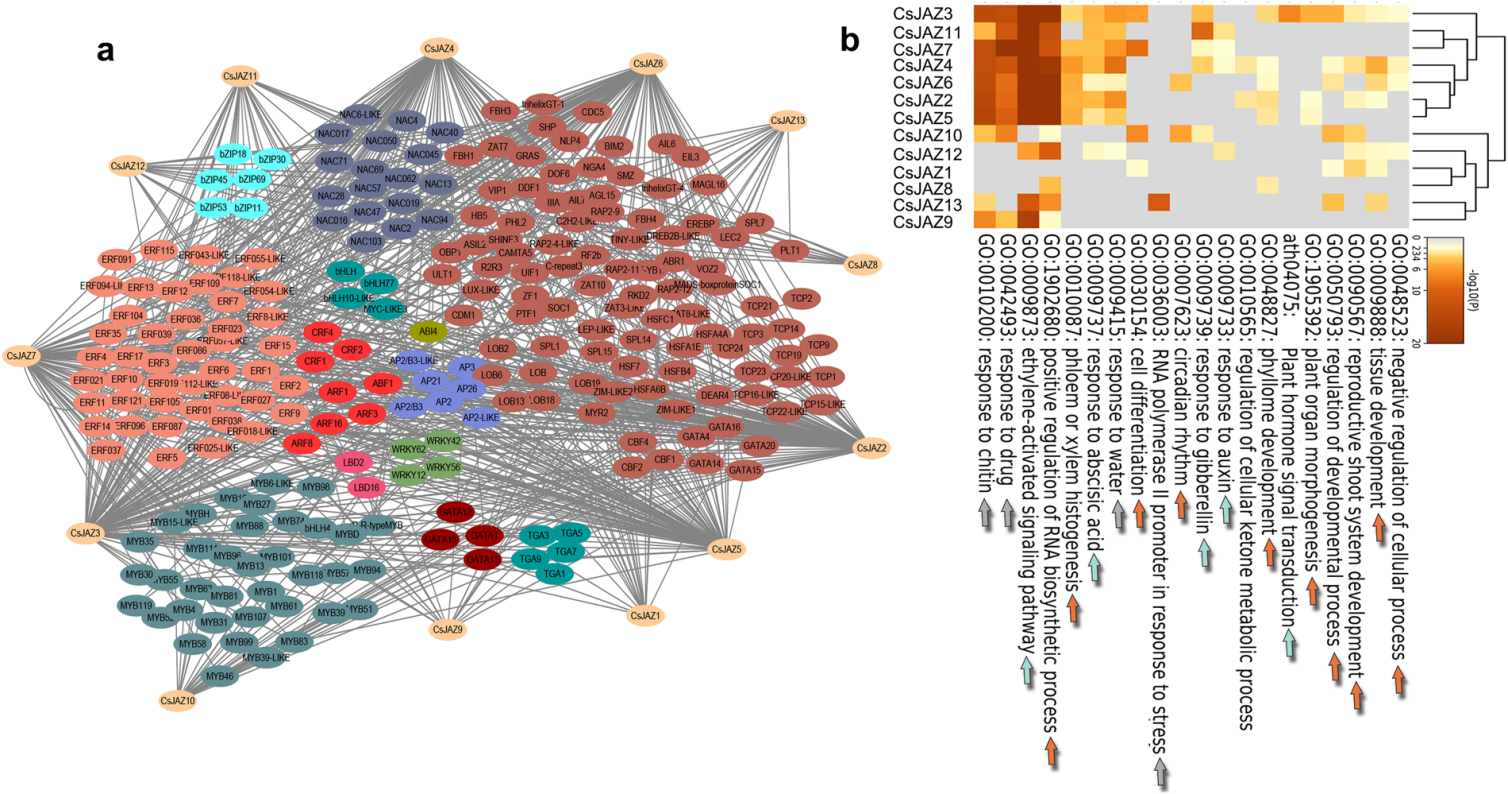
The putative transcription factor regulatory network of the *CsJAZ* genes. (**a)** The transcriptional regulatory network was constructed with the PTRM tool and Cytoscape 3.6 software. (**b)** The GO and KEGG enrichment analyses were conducted in Metascape. Orange arrows indicate pathways related to plant growth and development; green arrows indicate pathways related to plant hormones; gray arrows indicate pathways related to the plant response to stress.

To explore the potential regulatory network of the *CsJAZ* gene family, 13 *CsJAZ* genes were used for prediction analysis at the PTRM website. The results showed that the *CsJAZ* family was mainly regulated by numerous transcription factor families, including *ERF*, *MYB*, *bHLH*, *NAC*, and *TCP*. Among them, the *ERF* members were most abundant (42), followed by the *MYB* (36) and *NAC* members (18). In additon, numerous TFs related to plant hormones were identified in this study, including *ERF*, *CRF*, and *ARF* TFs. Many TFs involved in plant growth and development were also identified in this study, such as the *WRKY*, *LBD* and *AP2* TFs (Fig. 5a).

**Figure 6:**
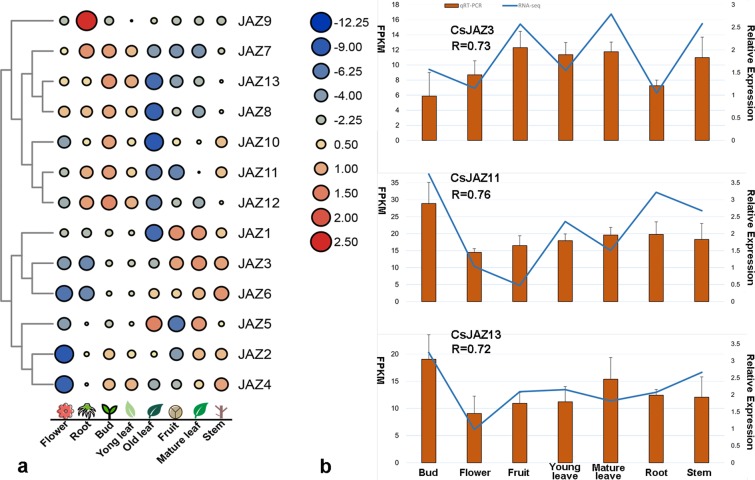
*CsJAZ* gene expression patterns in eight representative tea plant tissues. (**a**) Blue circles indicate low expression, and red circles indicate high expression. The circle sizes indicate different expression levels. (**b**) qRT-PCR verification of the RNA-seq results for three *CsJAZ* genes. The orange bar indicates qRT-PCR data, and the blue line indicates RNA-seq data. The correlation between the qRT-PCR and RNA-seq data was calculated with the Spearman test using the SPSS 17.0 software. Error bars indicate the standard error of the mean (n = 3).

To further understand the biological functions of these putative transcription factors, GO and KEGG enrichment analyses were conducted. The results showed that these putative transcription factors were mainly related to the plant response to stress, response to hormones and plant development (Fig. 6b). Morever, these putative transcription factors were mainly enriched in the ethylene-activated signaling pathway (GO:0009873), positive regulation of RNA biosynthentic process (GO:1902610), response to drug (GO:0042493), and response to chitin (GO:0010200). Among them, the ethylene-activated signaling pathway (GO:0009873) was most significantly enriched, and the highest number of *CsJAZ* genes was associated with this pathway, suggesting that the *CsJAZ* genes were involved in the interaction with the ethylene-related transcription factor (Fig. 5b).

### Expression analysis of *CsJAZ* genes in eight representative tea plant tissues

To further study the potential function of each *CsJAZ* gene family member, the expression patterns in the root, fruit, flower, mature leaf, old leaf and bud were analyzed (Fig. 6a). *CsJAZ1*, *CsJAZ3* and *CsJAZ*6 were highly expressed only in the fruit, and *CsJAZ7-8* and *CsJAZ10-13* were expressed most highly in the bud. However, *CsJAZ2*, *CsJAZ4* and *CsJAZ6* were repressed greatly in the tea flower. These results indicated that the *CsJAZ* family showed a degree of tissue expression specificity. Morever, we found that *CsJAZ1*, *CsJAZ8* and *CsJAZ10-13* were markedly inhibited in the old tea leaf compared to their expression levels in the bud, young leaf and mature leaf, implying that these six *CsJAZ* members might play an important role in tea leaf senescence. Interestingly, we observed that all *JAZ* members had similar expression patterns in the buds and young leaves, suggesting that the *CsJAZ* family might have the same function in these tissues. Finally, we verified the accuracy of the transcriptome data using qRT-PCR. The relative expression of *CsJAZ3*, *CsJAZ11* and *CsJAZ13* was measured in six tea plant tissues using qRT-PCR (Fig. 6b). The qRT-PCR results correlated well with the RNA-seq data, with R > 0.7.

### *CsJAZ* gene expression analysis in response to abiotic stress treatments and postharvest processing of oolong tea

**Figure 7:**
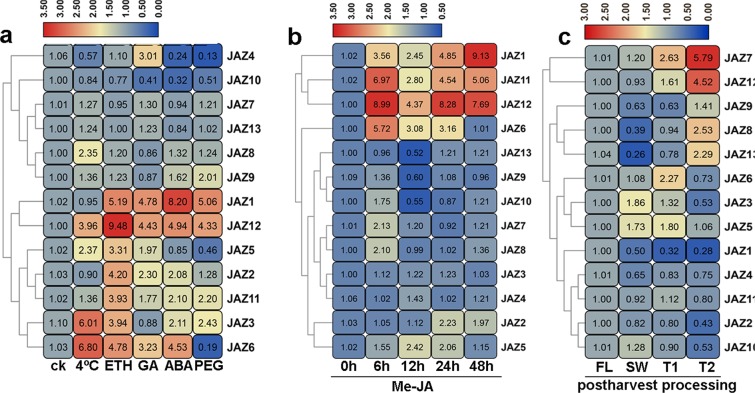
Expression patterns of *CsJAZ* genes in response to abiotic stresses and postharvest processing of oolong tea. qRT-PCR was used to determine the relative expression levels, and the results were analyzed using the2^−ΔΔCt^ method. (**a)** Expression patterns of the *CsJAZ* genes under six treatments as determined by qRT-PCR. (**b)** Expression patterns of the *CsJAZ* genes under MeJA treatment. (**c)** Expression patterns of the *CsJAZ* genes during postharvest processing of oolong tea. FL: freshly picked tea leaves, SW: sun withering, T1: first rotated, T2: second rotated.

To examine the responses of all *CsJAZ* genes to various abiotic stresses, including cold (4 °C), drought (PEG) and hormone treatment (ETH, GA and ABA), the expression patterns of all *CsJAZ* genes were tested by qRT-PCR. As shown in Fig. 7a, under cold treatment, *CsJAZ3*, *CsJAZ6*, *CsJAZ5*, *CsJAZ*12 and *CsJAZ8* were significantly upregulated, whereas the remaining *CsJAZ* genes showed no significant changes in expression. Under ETH treatment, the transcript levels of seven (*CsJAZ1-3*, *CsJAZ5-*6 and *CsJAZ*11*-*12) *CsJAZ* genes were significantly upregulated, whereas the expression of the remaining *CsJAZ* genes was not affected by ETH treatment. In response to GA and ABA treatment, the transcript abundances of five (*CsJAZ1, CsJAZ2, CsJAZ4, CsJAZ6 and CsJAZ12*) and six (*CsJAZ1-3, CsJAZ-6 and CsJAZ11-12*) genes were significantly enhanced, respectively. However, one (*CsJAZ10*) and two (*CsJAZ4 and CsJAZ10*) genes were significantly downregulated under GA and ABA treatment, respectively. In response to drought treatment, the relative expression of five (*CsJAZ1*, *CsJAZ3*, *CsJAZ9*, *CsJAZ11-12*) *CsJAZ* genes was significantly upregulated, but the relative expression of *CsJAZ2*, *CsJAZ7-8*, *CsJAZ10* and *CsJAZ13* was not altered significantly. Notably, *CsJAZ4* and *CsJAZ6* expression was inhibited. These results indicate that *JAZ* family members play an important role in tolerance to multiple abiotic stresses in the tea plant. Under JA treatment, *CsJAZ1*, CsJAZ6 and *CsJAZ11-12* showed a sustained up-regulated expression pattern. More specifically, *CsJAZ2* and *CsJAZ7-8* were significantly upregulated only at one time point, whereas *CsJAZ5* was significantly upregulated at 12 and 24 h. These results indicated that these *CsJAZ* genes were highly sensitive to JA treatment. In contrast, *CsJAZ9-10* and *CsJAZ13* were inhibited at 12 h, whereas *CsJAZ3-4* expression did not change among the five time points. Morever, we found that the expression of most *JAZ* genes at 24 and 48 h was not significantly different from that at 0 h (Fig. 7b).

During the postharvest processing of oolong tea, only two genes (*CsJAZ8* and *CsJAZ13*) were significantly suppressed at SW, whereas the expression of the remaining *CsJAZ* genes did not change significantly (Fig. 7c). The *CsJAZ6-7* expression level was significantly upregulated at T1, and *CsJAZ7* was maintained at high levels at T2, whereas *CsJAZ6* was induced at T2. Morever, although *CsJAZ8* and *CsJAZ12-13* showed no significant alterations at T1, the transcript levels of these genes were strongly enhanced at T2. Conversely, *CsJAZ2* was not significantly altered at T1 but was considerably induced at T2. Finally, the *CsJAZ1* expression level was dramatically suppressed from SW to T2. These results showed that the *CsJAZ* gene transcript levels were regulated by the processing treatments.

## Discussion

JAZ repressors have been universally reported to participate in extensive plant growth activities and stress responses and to play a hub role in hormonal crosstalk between SA–JA, ETH–JA and AUX–JA^7^. Eighteen *JAZs* have been found in apples^26^, 7 in sugarcane^27^, 18 in the rubber tree^28^, 15 in tobacco^29^ and 13 in tomatoes. However, the functional role of *JAZ* genes in the tea plant is poorly understood. To a large extent, aroma determines the price of tea products. Exogenous jasmonic acid spraying can effectively increase the alcohol and hexenyl ester contents in oolong tea and thus improve the tea quality^24^. In this study, we identified 13 *CsJAZ* genes based on the tea plant draft genome sequence (Table 1).

### The structural characteristics of the JAZ family in tea plants

The Jas (CCT2) domain mediates the interactions with COI and MYC, whereas the TIFY domain mediates the interactions with NINJA. In this study, all CsJAZ proteins contained Jas and TIFY domains at the C- and N-termini, respectively. Interestingly, with the exception of the TIFY motifs in CsJAZ5 and CsJAZ12, introns were inserted into all of the TIFY and CCT2 motifs in the CsJAZ family. Recent studies have shown that introns inserted into genes can serve as a buffer against accumulated mutations, which can confer some advantages for evolutionary conservation^30^. This possibility may explain why these two domains have been well preserved during the long evolutionary process. In addition, in terms of the number of introns, the *JAZ* gene family members contain 0–7 introns in some typical plants, such as rice, wheat and *Arabidopsis thaliana*^31^. However, 11, 12 and 11 introns were found in *CsJAZ2-3* and *CsJAZ5*, respectively. Large-scale research found that introns were no longer an energy burden in the eukaryotic genome and that the presence of introns had important implications for plants. In model plants, intron-mediated enhancers (IMEs), which are primarily located in the first ordinary intron position, can significantly increase gene expression. In addition, introns play an essential role in regulating alternative splicing (AS) and nonsense-mediated decay (NMD)^32^. Finally, introns also play key roles in mRNA export, transcriptional coupling, and other biological processes^33^. Therefore, abundance of introns in *CsJAZ2-3* and *CsJAZ5* may have some benefits. Notably, another non-mutually exclusive study suggested that fewer introns within a gene could allow plants to respond more quickly to environmental stress^34,35^. Therefore, *JAZ* genes with fewer introns may respond more quickly to the environment, such as CsJAZ6-7. Of course, whether the number of introns affects the function of *CsJAZ* needs further verification.

### Evolution of the JAZ gene family in tea plants

In this study, we identified a total of 96 JAZ proteins from 8 representative plants, including a bryophyte (*P. patens*, 7), lycopodiophyte (*S. moellendorffii*, 6), gymnosperm (*P. abies*, 13), monocots (*O. sativa*, 16 and *S. bicolor*, 15) and eudicots *(P. trichocarpa*, 13 and *A. thaliana*, 12). Remarkably, we did not retrieve JAZ family members from the algal genomes on the JGI website, which indicated that the JAZ family might have originated from terrestrial plants. In addition, we found as many JAZ genes in the tea genome as in the poplar, *A. thaliana* and *P. abies*, although the numbers were sharply higher than those in *P. patens* and *S. moellendorffii*. This result indicated that the JAZ family members in seed plants might have experienced an expansion event.

Next, all of the JAZ proteins were clustered into 13 subgroups using the ML phylogenetic tree analysis. We found some lineage-specific JAZ subfamilies. For example, the G8 subgroup only contained dicotyledons, the G9 subgroup only contained monocotyledons, the G10 subgroup only contained angiosperms, and the G5 subgroup only contained *A. thaliana*. The results show that the JAZ gene family may have undergone a lineage-specific differentiation event in the terrestrial plant genome. In addition, the JAZ genes from the gymnosperm *P. patens* and other angiosperms, such as polar, *A. thaliana*, *S. bicolor* and *O. sativa*, were clustered into the G13 subgroup, indicating that differentiation of these JAZ proteins might have occurred earlier than the divergence between gymnosperms and angiosperms. For the CsJAZ family members, CsJAZ12, CsJAZ1, CsJAZ13, CsJAZ8, CsJAZ7, and CsJAZ9 were directly clustered with three seed plants, whereas CsJAZ2-5 and CsJAZ10 were directly clustered with the bryophyte, and the G3 subgroup only contained one JAZ protein (CsJAZ11). This result can be attributed to the fact that tea plants have experienced a WGD event during their long evolutionary process. Thus, CsJAZ2-5 and CsJAZ10-11 may belong to ancient CsJAZ members.

### Transcripts of the CsJAZ family members are diffierentially expressed in eight representative tea plant tissues

The transcriptome data from the eight representative tissues were used to examine the potential functions of the CsJAZ genes in tea plant growth and development. The results showed that the CsJAZ family was differentially and constitutively expressed in tea plants. Similarly, two ZmJAZ members showed constitutive expression in multiple *Z. mays* tissues. In *Hevea brasiliensis*, eight JAZ genes showed higher transcriptional abundances in the leaves than in the bark. In sugarcane, the ScJAZ genes exhibited similar expression levels in the buds and leaves, with most of them showing relatively high levels in the leaves and buds. In this study, we found that the CsJAZ genes in young leaves and buds exhibited similar expression levels, suggesting that the CsJAZ family might have the same function in buds and young leaves. Moreover, in *Arabidopsis thaliana*, ZIM-DOMAIN4/8 (JAZ4/8) is involved in JA-induced leaf senescence. In the present study, CsJAZ1, CsJAZ8, CsJAZ10-11, and CsJAZ13 were markedly suppressed compared with their expression levels in mature leaves, indicating that these five CsJAZ genes might play an important role in JA-induced leaf senescence.

### The potential functions of *CsJAZ* members

Accumulating evidence has demonstrated that JAZ proteins directly interact with and repress many TFs to efficiently modulate various physiological processes^35,36^. Numerous JAZ-interacting transcription factors from different families have been studied in detail. In *Arabidopsis thaliana*, four bHLH TFs (bHLH3 -13, -14 and -17) could repress the JA responses by interacting directly with JAZ repressors, and the *bHLH3 bHLH13 bHLH14 bHLH17* quadruple mutant exhibited an obvious increase in plant defense^37^. OsbHLH148 interacted with OsJAZ proteins, leading to drought tolerance in rice^38^. In this protein interaction prediction study, four bHLH TFs were also found to interact directly with CsJAZs, implying that the bHLH TFs might redundantly repress JA responses in the tea plant. In addition, two MYB TFs (MYB21 and MYB24) were identified as the targets of JAZ1, JAZ8, and JAZ11, which regulate JA-mediated anther development and filament elongation^39^. JAZ4 and JAZ8 interact with WRKY57 to negatively control JA-induced leaf senescence^36^. Two APETALA2 (AP2) factors interact with four JAZ repressors to regulate JA regulation of flowering times^40^. In bananas, MaJAZ1 physically interacts with MaLBD5, which co-regulates JA-mediated cold tolerance of the banana fruit^41^. In wheat, JAZ proteins modulate seed germination through interaction with ABI5^42^. Similarly, a large number of MYB TFs, four WRKY TFs, two LBD TFs, and one ABI TF were identified as the potential targets of CsJAZ in this study, implying that these predicted targets directed by CsJAZ might be involved in anther development, leaf senescence and cold tolerance in the tea plant.

Moreover, our protein interaction prediction results identified a large number of TFs related to other phytohormones, such as ERF, ABF, ARF, and CRF TFs. In addition, the cis-element prediction analysis showed that most of the CsJAZ gene promoter regions had numerous hormone-related *cis*-elements, in particular many GA-, SA-, ABA-, and JA-responsive cis-elements. To further explore the functions of the *CsJAZ* genes in tea plant hormone crosstalk, the tea plants were treated with different hormones. The results showed that most of the *CsJAZ* members were significantly affected (|fold change| ≥ 2). For instance, *CsJAZ1-3*, *CsJAZ5-6*, and *CsJAZ11-12* were significantly up-regulated by ETH, whereas *CsJAZ1*, *2*, *4*, *6* and *12* and *CsJAZ1-3*, *6*, *11* and *12* were significantly up-regulated by the GA and ABA treatments, respectively (Fig. 7a). Similarly, in the tomato, the *SlJAZ7* and *SlJAZ11* genes were significantly upregulated by ABA^43^. In wheat, the transcriptional levels of *TaJAZ1* and 9 were significantly increased by GA treatment^44^. Moreover, the *JAZ* genes also exhibited distinct expression patterns under different hormone stresses in grapes and rice^45,46^. Therefore, we investigated whether the *CsJAZ* repressors might play a key role in tea plant hormone crosstalk. In addition, numerous studies have shown the important functions of the *JAZ* family in mediating JA-regulated responses. In *Arabidopsis thaliana*, eight *JAZ* genes (*AtJAZ1-2*, *AtJAZ5-6*, *AtJAZ7-8*, and *AtJAZ9-10*) were responsive to JA^47^. Consistently, the *CsJAZ1*, *CsJAZ7-8*, and *CsJAZ12* expression levels were significantly increased at a minimum of one point time by MeJA treatment (Fig. 7b). Previous studies have shown that the MeJA-induced gene up-regulated was controlled by short transcriptional cascades^48^. When a large number of JA-lie bioactive are accumulated in the tea plant cells (under Me-JA treatment), JAZ repressor protein will be ubiquitinated by SCF COI1, which affects the expression of Cs*JAZ* genes. On the other hand, some specific JA-regulated TFs may involve in the activation of *CsJAZ* gene expression under JA treatment. In addition, some cis-elements related to JA response exist in the upstream or downstream of the *CsJAZ* gene may also be responsible for the gene activation.Interestingly, we found that *AtJAZ1-2* and *CsJAZ12* clustered into the same subclade, *AtJAZ7-8* clustered with *CsJAZ7*, *AtJAZ9* clustered with *CsJAZ1* and *AtJAZ10* clustered with *CsJAZ13* and *CsJAZ8*. These results suggest that the five *CsJAZ* genes, similar to their homologs in *Arabidopsis thaliana*, play an important role in the JA response.

Previous studies have indicated that JAZ proteins are involved in the ICE–CBF/DREB1 transcriptional pathway to positively regulate the response to cold stress. In apples, overexpression of *MdJAZ1* or *MdJAZ4* weakened the promotive effect of *MdMYC2* on cold tolerance^49^. In wheat, most of the *TaJAZ* genes were highly sensitive to drought and low-temperature treatment^44^. In this study, *CsJAZ3*, *CsJAZ5-6*, *CsJAZ8* and *CsJAZ12* expression was significantly up-regulated by cold stress, suggesting that these *CsJAZ* genes might be involved in the ICE–CBF/DREB1 response to cold stress. Consistent with the evidence that JAZ from rice and grapes could be induced by drought stress, eight *CsJAZ* genes significantly responded to drought stress, which implied that these genes played an important role in the response to drought stress.

### Expression and functions of *CsJAZ* genes during postharvest processing

Previous studies have reported that terpenoids and green leaf volatile-related genes contribute significantly to tea aroma formation during tea processing^50^. During the oolong tea postharvest processing, the rotation and withering processes, which can be regarded as light and mechanical wounding stresses, induce the up-regulation of many genes related to secondary metabolism. In fact, numerous genes related to secondary metabolism are thought to be involved in plant defense responses against damage stress based on the JA-dependent elicitation of their biosynthetic pathways^51^. In this study, first we comprehensively investigated the expression profiles of the *CsJAZ* genes during oolong tea postharvest processing. Most of the *CsJAZ* genes were significantly affected by these treatments, implying that the accumulation of secondary metabolites during tea processing might result from changes in *CsJAZ* expression levels. Hence, in the future, many genetic technologies, such as RNAi and CRISPR/Cas9 may be used in the breeding of high aroma tea varieties on the basis of some functional *JAZ* genes in this study. Definitely, the role of *CsJAZ* during tea processing needs to be further determined by measuring the endogenous JA content and accumulation of secondary metabolites during tea processing.

## Conclusions

In the present study, 13 *JAZ* genes were identified based on the tea plant genome for the first time. Some potential functions of the *JAZ* gene family were revealed in the bioinformatics analysis. Interestingly, we predicted a large number of CsJAZ-targeted transcription factors, some of which were verified in other plants. In future studies, we will further validate that *CsJAZ* targets TFs in the tea tree using experimental methods, such as the yeast two-hybrid assay, which will help provide more powerful evidence to reveal the functions of the *JAZ* family in tea plants. The *JAZ* genes are key repressors in the JA signal transduction pathway and plays an irreplaceable role in plant leaf wound stress responses. In this study, our results showed that most *JAZ* genes are dramatically up- or downregulated during the tea postharvest processing, indicating that the JA may play a certain role in the formation of aroma in tea processing. Try to breed JA sensitive tea plants and overexpress or inhibit the expression of the *CsJAZ* gene in tea leaves may be a new clue for the breeding of high aroma tea varieties. In future studies, we will further explore the role of JAZ in tea processing via transcriptomics and metabolomics.

## Materials and Methods

### Identification of CsJAZ genes

To accurately identify all *JAZ* family members in the tea plant, three different screening methods were combined. Firstly, a total of 13 annotated *CsJAZ* genes (K13464) were obtained from the “Plant hormone signal transduction” KEGG pathway in the tea plant genome database (http://pcsb.ahau.edu.cn)^52^. The BLAST algorithm was used to identify potential tea *JAZ* genes using all *AtJAZ* genes from the *Arabidopsis* genome database (www.arabidopsis.org) as queries (BLASTP, E value ≤ 1 × 10^−5^). Next, the HMMER software was employed to identify the predicted tea *CsJAZ* genes with the Hidden Markov Model based on the TIFY (PF06200) and Jas domains (PF09425) (hmmsearch, E value ≤ 1 × 10^−5^). Finally, CCD (https://www.ncbi.nlm.nih.gov/cdd/) and SMARAT (http://smart.embl-heidelberg.de/) were used to verify whether all potential tea *CsJAZ* genes had complete TIFY and Jas domains. The physical and chemical parameters of all *CsJAZ* genes were predicted by ProtParam.

### Phylogenetic analysis of CsJAZ proteins

The JAZ protein sequences from Physcomitrella patens, Selaginella moellendorffii, Sorghum bicolor, Oryza sativa, Populus trichocarpa, Arabidopsis thaliana, Picea sitchensis and Camellia sinensis were downloaded from four public databases (JGI, https://genome.jgi.doe.gov/; TAIR, https://www.arabidopsis.org/; TIGR, http://rice.plantbiology.msu.edu/; and NCBI, https://www.ncbi.nlm.nih.gov/). CCD (https://www.ncbi.nlm.nih.gov/cdd/) and SMARAT (http://smart.embl-heidelberg.de/) were used to verify whether all potential JAZ genes had complete TIFY and Jas domains. Finally, a total of 74 JAZ protein sequences were subjected to multiple alignment analysis using ClustalW in MEGA-X with the default parameters. The phylogenetic tree was built using MEGA-X with the maximum likelihood method and Poisson correction model. Bootstrapping was performed 1000 times.

### Exon–intron structures, conserved motifs and promoter analysis

The percentage identity matrix of the *CsJAZ* genes was built using the DNAMAN 7.0 software, and the results were visualized with TBtools. The CsJAZ exon–intron structure information, domain location information (Jas and TIFY domains) and 1000 bp sequences upstream of the initiation codon (ATG) were obtained from the tea plant genome database. All CsJAZ protein sequences were submitted to MEME (http://memesuite.org/tools/meme) to analyze conserved motifs with the following parameter: maximum number of motifs, 10. Cis-acting regulatory element predictive analysis was conducted in PlantCARE (http://bioinformatics.psb.ugent.be/webtools/plantcare/html/)^53^.

### Prediction of transcription factor networks

Transcription factor network prediction was performed as described by Wang *et al*.^54^ with minor modifications. The *CsJAZ* CDS nucleotide sequences were submitted to the Plant Transcriptional Regulatory Map (PTRM) website (http://plantregmap.cbi.pku.edu.cn/regulation_prediction.php) to predict the target transcription factors of the *CsJAZ* genes with the following parameter: p-value ≤ 1e-6^55^. The Cytoscape 3.6 software was used to visualize the transcription factor regulatory network^56^. Then, the predicted TFs were subjected to KEGG and GO analyses using the web program Metascape (www.metascape.org)^57^.

### Expression profiles of eight representative tissues based on transcriptome data

Raw RNA-seq data, including young leaf (SRX4343640), young root (SRX4343634), young fruit (SRX4343633), apical bud (SRX4343639), flower (SRX4343636), young stem (SRX4343635), old leaf (SRX4343638) and mature leaf (SRX4343637), were obtained from the SRA database^58^. All clean reads were mapped to the tea plant genome using the TopHat2 software^59^. The gene FPKM values were calculated using the HTseq software^60^. The expression profiles were visualized using TBtools.

### Tea plant materials and treatments

All tea plant cultivars (*C. sinensis cv*. Tieguanyin) were planted in an experimental farm at Fujian Agriculture and Forestry University (Fuzhou, China) under the same cultivation conditions. To verify the accuracy of the transcriptome data downloaded from the SRA database, eight representative tissues (young leaf, young root, fruit, apical bud, flower, young stem, old leaf, and mature leaf) were collected from three-year-old cutting seedlings of the tea plant cultivar. The tea plants were treated as described were previously^61^. Briefly, the tea plants were treated with the following conditions: drought stress (10% (w/v) PEG-6000), low temperature stress (4 °C, 6 h), hormone stress (ABA (100 µM, 6 h), GA (100 µM, 6 h), ETH (100 µM, 6 h), and MeJA (100 µM, 24 h). Fresh second leaves from the untreated plants (control) and plants subjected to the different treatments (drought, low temperature, ABA, GA and ETH) were harvested at 6 h. Plants treated with MeJA were harvested at 6, 12, and 24 h.

For the postharvest processing analysis, the oolong tea manufacturing process was followed based on previously reported methods with minor modifications^62^, freshly picked tea leaves (FL) were exposed to sunlight for 40 min for sun withering (SW) and subsequently rotated two times (T1 and T2) at 90 min intervals. Samples at every stage (FL, SW, T1, and T2) were immediately collected for further analysis in this study.

All sampled tea leaves with at least three independent biological replicates were frozen in liquid nitrogen and stored at −80 °C.

### RNA extraction and quantitative real-time PCR analysis

Total RNA was extracted from the tea leaf samples using the RNAprep Pure Plant Kit (Tiangen, Beijing, China). The quality of the extracted total RNA was checked using the NanoDrop 2000 spectrophotometer (Thermo Scientific, USA). The cDNA used for quantitative real-time PCR (qRT-PCR) was synthesized using the EasyScript One-Step gDNA Removal and cDNA Synthesis SuperMix Kit (AE31102, TransGen Biotech, China). The qRT-PCR was carried out using the Trans Start® Tip Green qPCR SuperMix kit (AQ141-02, TransGen Biotech, China) and was performed on the Bio-Rad CFX96 Touch™ Real-Time PCR detection system. The amplification was performed as described by Wang *et al*.^61^. The relative gene expression levels were measured using the 2^−ΔΔCt^ method. All specific primers are listed in Table S1.

## Supplementary information

Supplementary information.

Dataset 1.

Dataset 4.

Dataset 5.
