# Supplementary material for: Genome-wide and expression pattern analysis of JAZ family involved in stress responses and postharvest processing treatments in Camellia sinensis
Source: Sci Rep. 2020 Feb 17;10:2792. doi: 10.1038/s41598-020-59675-z (PMC7026426; doi:10.1038/s41598-020-59675-z)
Supplement: Supplementary file 1 — Supplementary information. [file 41598_2020_59675_MOESM1_ESM.pdf]

Genome-wide and expression pattern analysis of JAZ family involved in stress responses and postharvest processing treatments in *Camellia sinensis*

Yucheng Zheng, Xuejin Chen, Pengjie Wang, Yun Sun, Chuan Yue, Naixing Ye*

College of Horticulture, Key Laboratory of Tea Science, Fujian Agriculture and Forestry University, Fuzhou, Fujian 350002, China

Correspondence: Naixing Ye, ynxtea@126.com

Table S2. Full-length protein sequence of JAZs in tea plant.

>CsJAZ1

MERDFLGLNSKDSVVVVKEEAVEGCKDSAFSKGSGVQWPLSNKVSALPHFMSFKVAQDEMKPKVMSDPL
AASGFMSISTADAFDTTRKHRSGETQKVHDGQGGVHFSMTNYPVQHNALPMHFSHDVKMLPAANSMSNP
FFKTHFSGAAQNFAGATMKQQLLGGIPVTAPHPMISSLASVAGTTEPWLNSKSSEAPPQLTIFYAGTVNVYN
DISPEKAQAIMFLAGNGASNKAQPRAQVQVPTSKQTGGDGVLGNQPTINTPPNSGLSSPISVSSHPVGQSGN
GLTANGEMMAAKPNGVSTTPLSKIDPPKIGPLAANTMIPSVGQSGNGLTANGEMMAAKPNGVSTTPLSKID
PPKIGPLAANTMIPSAIPQSRKASLARFLEKRKERALNSAPYNVSVKSPECATPGST

>CsJAZ2

MEAANPQPLQARPYEEHAQVPAIQIEDDDDGEYEDGVDGGGDDAMDEAEEAHVTSVNASDHHRHGGGD
GGSGGVVVASRTSELTLAFEGEVYVFPAVTPEKVQAVLLLLGGREIPTGVPTIDVPFDQNNKDLDDNPKRSN
LSRRIASLVRFREKRKERCFDKKIRYNVRKEVAQRMHRKNGQFASLKKSPGAACWDSPNSCPQEDSTPRPE
TVVRRCHHCGVSENSTPAMRRGPAGPRTLCNACGLMWANKGTLRDLNKGGRNLSLDQIEPGTLRDLNKG
GRNLSLDQIEPETPIDIKPSIMEGENFFGNQGDPGTPEDPSRAVTGGTDNPSLNPDEEDTAEDHPNSSPMGIV
NSSCNLDEQETLVELASASETEMEIPANFV

>CsJAZ3

MPETNHEASMYVAEAMNMQQQQQQNQIEDEEDDEVAGGESIDNPQIRFEAHAALHDGGGGPVMNGVDG
VHPHALYVPGSEIPPAVGGGGADQLTLSFQGEVYVFDAVSPEKVQAVLLLLGGYEVPTGIPTVGVAPQNQRG
LADFPGRSSQPQRAASLNRFREKRKERCFDKKIRYNVRKEVALRMQRRKGQFTSSKTISDEVGASSDWNAS
QGSGQEEQETLCTHCGISSKSTPMMRRGPAGPRTLCNACGLKWANKGQFIRSIVSWNSDTICSFYFGIGSTM
LGLGCSQILLCGSCMKVRLEHEVASQYVTGAPMVSSFLFPYSHALLLDFLLISSCEGVLRDLSKVSAAGIQD
PTVKAAEQSEGEANDSDVVTSAADIINSSNGENSAVPAERPNLGVSREAGLMKRNNVETSSAKRCNVRTIK
LVVVSEEKGAAMVSKGVTVGSGTAGRAKEDGREPR

>CsJAZ4

MYAHSQSLNVPNHFTTADQDPSPCAGGGGGESAADNPHVSYETHSLNDGVGIDDVHIVPSDVVYADGASD
LPLQRTDCSNQLTLSFRGQVFVFDDVTTDKVQAVLLLLGASELSSTMQGGELPYENQRESMEYPVRCSLPQ
RAASLSRFRQKRKERCFDKKIRYNVRQEVALRIVRFVIQIVRFCSIHLEINSNRLMESQMAQNRIRIARFRIVA
GPRNLNANRGKRKIFEDDEIELIEGGEEEEEEFDEVTMADDDEEDDSDIDLGDDED

>CsJAZ5

MEAVNPQPLQALPYEDHLVFPTEDDGDDYDDGGDGDDGMDEAEDEHVTSVNASDHHHHYYGGGGGVV
VASRTSELTLAFEGEVYVFPAVTPEKVRSSSIGLVQAVLLLLGERKIPTGVPTIDVPFNSNNRGVDDNQKCSNI
SRRIASLVRFREKRKERCFDKKIRYTVRKEVAHRMHRKNGQFASLKENSATSSWDSTKSCLQGDGTPRPET
VVRRCQHCGVGENSTPAMRRGPAGPRTLCNACGLMWANKGTLRDLSKGGRNLCLDQIEPETPVDIKPSIIE
GENFCGNQDNPGTPDDPSKVVTGETGSPVNPDEEDLCGIAEDHTNSLAVGIINSSCNLDEQETLVELVNICSE
TEIDIPANFSVDFFKRKSKQELVGASNGE

>CsJAZ6

MPETNHQSSMYGTEPTILQQQQQQMNQIEEDEEDDEVVGGESIDNPQIRIEAHSLHDATVGAGSVAVAGAG
DAMNGGVDGVHPHALYVPGSEIAPAAAGGGADQLTLSFQGEVYVFDAVSPDKVQAVLLLLGGYEVPTGIP
TVGVAPLNQRGLGDFPVRSSQPQRAASLNRFREKRKERCFDKKIRYNVRKEVALRMQRRKGQFTSSKAVS
DEVHSSSVWTTSQGSAQEEPETS

>CsJAZ7

MKMRRNCNLELRLVPPSAPLRPADYNHHHRLHHFQPTLDLLGGSPNERQQEQPLTIFYNGMVNVSDVTEL
QARAIMMVASREMEEKLKSLLGPEPSSPLQQSPLCSPSTGLSMKRSLQRFLHKRKNRIQATSPYNH>CsWOX

7
MSPAGSSRWCPTPEQLMILEEMYIGGMRTPNASQIQQITAHLSLYGKIEGKNVFYWFQNHKARDRQKLRRK
LTKHHHHHHQNLQSPPPLPSQHSSIGFLPQGGEVEDASTQMVTSKPMVMRSYGRDWMVMLDVVPTPITPC
CNTAPPKTLELFPITATGLKDMHFPSSMSSN

>CsJAZ8

MSRSAVELDFFRMEKESSSKKFIDRRRSFRDIQSVISKLNPQLLKTVIASGSVDHTYNNVAKSSENCNLFLSS
KSSFSVPSTPQHEDTLVFPPALPLYSPAFGSNASGSEPTPETTPMTIFYNGSVTVFDVPRHKAENIFKLAEKQV
SKTVESAADPKLALSSTDKTQILDTINGDLPIARRKSLQRFMEKRKERLTFVSPYGCPNDYASSGQNTSGQR
E

>CsJAZ9

MSCSSVVAKDFMKIEDIAQIEEELDYQKENLKSSEDKTQTLDLFRKYLVPRTPHNFGKTNEEESGAIKRATL
RRFVATKICNGSRPPSFLEKALLPGISGNATVQTSPSGAKASAAQLTIFYGGQVNVYDDVPQHKAQAIMVFA
GESSLSAPISSNVPKKEVRKPLHRPNLPSVCKLQADLPIARKYSLQRFLERRFRRALKSPYAHTDTRNKDEN
DHNHDHHDEAIATSHDLMIMNEKGGSFTPSPFPSRLGYSLHKERC

>CsJAZ10

MDNSIHGSLHNVHYLQEHDDDHHHDVLQHMRNGNGLDNNEHDDNDGNGGESEGMEGDIQVHPDTRNLS
DNQNGLVARGGNENNNNQLTLSFQGQVYVFDSVSPEKVQAVLLLLGGREIPPTSMPTVPISTHQNTRGLSST
PEHCSVPHRLASLIRFRAKRKERNFDKKIRYTVRKEVAIRMQRNKGQFTSSKPNHDDCASAVTSWDSNQSW
GLDGSGSQQQEIVCRHCGISERSTPMMRRGPEGPRTLCNACGLMWANKGTLRDLSKAAPQLGQNPSLSQN
ENGNFDADQMVCRIAGNGSDSS

>CsJAZ11

MERCFLGLGSKNKSVAVKEGITVARKDSGVGREADKDGRRDQRCEEHCEGLKWRLEHSMAALFSLPLPLP
SPYFSHDAKLSLVAKNGYQSTIWTEIAASGASGMQWSFSNKVSASPQYLSFKANQDDGGPRKTGFDSLSST
RFMTISSAEAFESNHKLYSSIVQKNLNLDKQAGTHYTVTSYPPKQFDAHSRPHEARMLPMANQTNQKISIIS
SAINPQPLRAVPVISSVSVVPTSTSVVGTTDLRNSSKPSGGPAQLTIFYAGSVCVYDNVSPEKAQAIMLLAGN
GHSSTPNKAVATTQVEVPVPRPSGVDNFIGNQSRTTSPCLGLPSPISGGSSSSTAITAVRAIGTLASTSNKSEPS
TGVSSRAPVSAPLVPSAVPQARKASLARFLEKRKERAMNASPYVSKQSPNCSNPGLGSLSFSMNTDGSSPL
QAII

>CsJAZ12

MSSSSGSADSGRFSGHRHSSRVPEKSASSSFSQTCSLLSQYLKEKKGTFGDLSLGMPTATTTMNLFPVAMKS
SEVSRNGTPVTRNLTSMDLFPQQSGFGSNISVEESLNKVDSSAINKSEPETAQMTIFYAGQVIVFNDFPADKA
KEIMLLASKGSSHHNPGTLASTPVQKPIEPTNLIPTSSASAIVPNSGNNTISIEPISLIPTTSSVAAPNFSNNMIQ
ERVQVQRASQPIATDLPIARKASLTRFLEKRKDRITSRAPYQISSSTSPPKPTESKSWLGLAAQSPVQFEHHL

>CsJAZ13

MASRSAVELDFFRMERDCSSKPLPNKLFDRRRSFRDIQSFVSKMNPELLKTVIASGSVDQSTNLSKSSSFSVP
VTPKHEENVFPAFPLYTPTFRSNTCGSDQSTPESGPLTIFYNGTVTVFDVPRHEAENILKIAKGGVSKTVESV
NPELALSSTDQHQLLETLKGDLRQTRTKSLQRFLEKRKERLTLELSPYGCPTRNTFSGKNTSRYED

Table S3. The sequences of JAZ proteins in different plants

>PsJAZ1 ***Picea sitchensis***

MSQDQAVAMDFLGVEKGSVKKESQTDENPVTAQSKQEPVATEQMTSPAASAPSEGSTMNFSQAFSLFSKHFNEKGNGNA
AIGRGENVYLGNGKEDVKGSKPVNEENVSTQLGLFSQQSGFGSLRPFPGAEQKGFASTTAQPWLFRGILPPNSPAMRPAVT
STPPIKPPTAQLTIFYAGMVNVYDDVPADKAQAIMLLADSGNPLNASFIKPANFAQQTPCVSPVSSPLSSIPRTSLSLQTSGV
TTEVATPTMPLAPNHHQPIRKLQADLPIARKHSLQRFLEKRKDRLMTKAPYPASPAPMKSEMEESVSASPLLLSCQSPSSLT
SRPLSGVCCNENSVTQEPSPPPVSAYIS

>PsJAZ2

MERDSVVARDFLGLSGGKDISRGSNGSESKSSTSAAQVQAQSQAQQREEMKPYLKDNGVCLANISLQWPYSNKPAALEQ
FISFKQEGPKKSSFDQLSNSGFRPISTADAFDLINKSAPGMQKNFNHDPMARNTIESGNYYSGQNGALSRHFGLSHITYSA
HPFDEQGSSGYNMQKVGTFPCSNHSLSVSNLGSPAYFKGEQAAGTTAPLMRQFQGMPLAGQHSFLPVVGASASTTIPGAV
ASVSEKPAGAQLTIFYAGSVNVYDDVPADKAQAIMFLAGNGNFWSGKTATPPSQSPAMPISVATNGHARQATPIGPRLSNA
LSPTNPQVHSQNSHATVQTSQSITAISSAGDSQTNQNNGASSNLHETPKVNAAVSTPAPIMPRAVPQARKASLARFLEKRK
ERVCTKAPYQTKKSFDDSSHGEVPLSPTRSSTPLVDGYKSQRNEFMPRSEEKASCLGKEQHEQPWPPKVEKNEIKECQLQ
LT

>PsJAZ3

MESRMERDFMGLGRRNGNEMESEKTSNNEQPHGEREKIMSLSLFQEAEKSTKLQLSSGSFCGLSEAIGKAGIANGSGKVS
VPNTSMQWTYWTKAAALQQFMSFKNYQEERPEKRKFNQLVSSGLQPVATVDTFKSNHRSTSASQKSCNFSPISKDNAEF
RNGYSGTNCVPNQHFEVPPANFSVKAFDDQSFCNSSIEQAGAFLREKASHSPPNFREHFSSPASFKGQDSTAALGGKLLSSI
PFTRQAPVLCGAAASTVGTTPMRADSNLPGKLASAQLTIFYAGTVNVYDNIPPDKAHAIMLSAENGSSMSTKMTDLSPQN
FVMSTPVPTNSHSRPATPTICSSRPNALTSMIPQGPPQKSHMPIPQVHPQKSQICSKPGQSTGVSSSNGELKSNQNNGAGSN
QQEGAKQMVSASTPNGVIPRALPQARKASLARFLEKRNGRVTPNLSSPTEKSPHHSSLQQEKSFSSMSSFDGCSSPQHDVT
SDALHMKKLSRSDSMAHANHLTLQKAGFMLEERKIAFGTRNC

>PsJAZ4

MKNDKGKRPLEERSFRESAEENSIKGMADLIPDYLGDTYSSFTYRGSPTPAEKRDLEVCNPCRAASNGRVTKSMATVKTG
IYELPNLDLSLLPPRWPAGTGSSPYQRGVQPAASAQQYSQVPSSPNPDVFRSPNAGAEQPPCAQLTIFYAGTVSVYNVSAD
KAKSIMMAASRICGSPHSGSPSSSASTPSTQLNPTPRSMDMSPISSDSAPVAILNHTQASETIKTAAIPISRKCCIQRFLEKRN
ERIHGKSPYLPPAQN

>PsJAZ5

MSPSASVAFEYLRFQNASEANETVLNGSNSLEFSPGKHQAKPRLMQDLSRIKPQMMLKLLSSNKLSEDTKLDFAHVNRQ
AEGFNKQSEATSHAKMIELFPVSTGFGSDPRVTETKITLPLEIGLTDPNSSRCVLAEVPAKPSTAQLTIFYNGAVNVYDMPAE
KAQEIMKLASANSSSNTRISTITSSKIEQILQHQPSKPALNAINENQPQRLAVGMEIVMKLSLQRFLQKRKERLNSVAPYST
METETSPWKAGKDSDDQIILSLGCP

>PsPsJAZ6

MSTTASVALEFLRFQNASEANGTVVNGCTSLEFCPGKQLAKPRSVMPDLSRIRPQMMQKVLSSIGLSEDKKAKSFNGQSE
ASSHTELMELFPVSTGFGSNPRVAETNITLPFATGQTDPDSRSVPAKAPEQSSTAQLTIFYNGAVHVYDVPAEKAQAIISFASS
NSSINTGTSPTTSSQIEQISKPFPSKPPSKPQSNAVNEKQTHRPPIGLEIVRKLSLQRFLQKRKERINNVAPYSSMKTATLPSK
VEKDTADQIFLSLAGPTQHF

>PsJAZ7

MASAFEEHCNQEKGLPSWIKTVNGGAKRAAADFIPRLDLSSVGSCFNSIHLFPNQSGFGSPIAVLPDSEHRTSSDDHQDGC
SGFVTSFRPAASTIPSKQSCAQLTIFYGGVVNVYDDIPADQAQAIMLIASSENYSGYPHTKVQNSTCRSQTELKTSLPVMKF
SGESDLPIGRKHSLQRFLKNRKERVIANAKSPYTAADAANTPKGLRSNSPPESHPPP

>PsJAZ8

MSTSAAVASEFLRFQNASAANGTVANGSTSWTFVPQKQLAKPRFMQDLSRIKPQMMQKVLSSIGLSEDKNADFALVNRQ

AEDFNRHSEASSHREMLELFPISTGSGLGSNPRVTENKITLPFAIGQTDPDSSSSVPAKAPEQQSTAQLTIFYNGAMNVYDV
SAEKAQAIMRLASANSSRKTRISTISSSKIEQVSKPLPSKPASNAANGNQPQTPPVGLEIAKKLSLQSFLRKRKERFNSVAPY
TTMKPVTLPSKAEKESDDQIRLSLASSSQNFKMSVNAIDLNA

>PsJAZ9

MSRGQGLVTMDFLGIDKSKKNASEIKYCTPGLIEKSIHCIKPQMVQQAFSSRRLCEEYCKASRDSEDLKFEAASRIVKELFP
QHTARGSIPAAKHNIATTMGNSSICRSAYTTPPGEPSTSQLTIFYNGTVNVYDVVEKQAENIIRLASNAQAEDIIRLASNEISC
QQLRSVGFDRHTLSKTSTIWSTNTNNQSEEPLPLNPSKNSTPRQVVIQKLNTDLPLQRKQSLQRFLQNRKEYRMNRAAPY
SHSHTQSSLKKGSKLRPDCELIDCDEHHFLEKN

>PsJAZ10

MSPSESVAFEFLRVQNASEANGAGVNGSTTLEFGPGKQLAKPRLQDLWRIKPQMMQKVLSSIGFSEDKKTDFAHVNHQA
KGFNGCSEASSHAEMMELFPVSRGFGSNPGVAEKNITVRSAISQTDSVSRSVPAKAPEQQSTAQLAIFYNGMVNVYDVPP
EKAEAIMRFAGDNSLNKTSTPKINSCKIKQILKPLPSKPASNADNEDQPERHPVGLEIVRKLSVQRFLQKRKDRINSVAPYT
TMNTATLPSKAWKDSEDQIILSLACPSQ

>PsJAZ11 MGLVVSTKLKLGTAFEEQCDEETGLPSWITSVNGGAKRAVADCISRLAPSMIPEKPSSAKLSIFYGGTVY
IYDDIPTDKAQAIMLMASSGNYSSYPHTKVHNGWGSQTEQKLSVPVIKLSNGSGIHPQTSSPKLRTGSSDIPIARKHSLQRF
LQNRRDRVNANAKGPYKTAEAERSSIVQSECKRKVSI

>PsJAZ12

MQRVKIVERDFMGLINSEVVAEKDNEGLTKDSNTDTNLEDGSDSANKAPCQHFGVSSVAHSTGSIDGLSSSAHHIDEAGG
FPSSSLSLSMSNFREHLGFNAISKGLQATSTSCVTTSMVNPSAIQKNSFFPAAAGVYPSSTATSNATAVPGKQPCTQLTMFYA
GTVNVYDNVPVEKAQVLMLLAASARSKKMTNLSPRSSAMPTSIATAVHAMPITSPFSSKQPNRVFPMTSQAPLQKAQASI
QPNPYSAVTLNADDSQIIQSIATSSNQQEASKSSDAGITPAPLISRAVPQARKASLARFLEKRRERIITKAPYPTKKSPDCSPQ
REESPSSKHIPPPLDGCLTKQNQLISKGLDEKFPIDSESMGSFLDKDQKEHSCSLKVSCKEMGECETTVEASIL

>PsJAZ13

MVKVVAAAMDFLGILSEKEKSELTKNGSNSEGIQKDQTEMNAVVTASTDKAQWNNNNAMRSDGSFKNRFCEKGSFTSQ
LRDGLAFEEHCDEEKSFPSWIRTVNGVAKRSVADCIPRPLDLSVVGSSSNLINLFPDQSGFGSPIAVKGGFDHRTSDNDGDG
CSGFVTSFRPTASMISGKQSGAQLTIFYGGTVNVYDDIPADKAQAIMLIADSGNHSSYPQTELQKDCRSQITEVKISPLPLV
KLQEGSRIHHQPASYKMYTDLPIARKYSLQRFLEKRKNRLNANAKSPYSTAAEADNTKPRRYPSSSSAIPLHSNCPR

>SbJAZ1 *Sorghum bicolor*

MAPSPMAQAGCSGRRRFAVACSVLSLCVRAETAAAAAAAHSRSVAQAQAGAASPMLLMPGADVVSDETVPTPAPAQLTI
VYGGRVQAFSDVSADRAAEVMRVAARQDMPPAGGLADLQVARKASLKRVMGKRRDRLGARAPYAPARPPPPKEQQEK
DADIWLGLGISG

>SbJAZ3

MATAAGSIQQGHGARFAAACDVLSRYVKAAAAATTTTPVEQPRPLPGTVVGVVLPLMPGADLSTQAEELAEAADQAGP
APRAQQLMTISYGGRVVVLDDVPADTAAELLRLAAAAAAPRVLRAPDDLPMARKASLRHFMEKRKGRVAKRASPYSRP
GDAGAAAASSFPDHLALSL

>SbJAZ5

MAAAAPWSGSNTATTTTTSRFAAACGALSQYVKAAEAERTTRTRPPAAVRPLPLMPGADVDQDHDEQPETAAAQLTIVY
GGRALVLDDVTADKAADLLRLAAAARGGTEQLPLSSVADLLLPVARKASLQRFIEKRKDRVAAGPVTHATISRSRCDLTK
RVSALARGCCAEEAQLSRWGIEISDSV

>SbJAZ9

MAAMPTDSMTRRFAVACGVLSQYVRNGAPATTMTPPPPFLKQAPAAGPAAAQEMIVASASAPQQLTIFYGGRVVVLDACP
PEKAAELIRLAAAAAQGGSTQPPEQALVDMPIARKASLRRFLAKRKDRSSSASPAPYDDRQDDDEPPAPKKGKMAAAAT
REEPSSSSWLALGSLCSMHSR

>SbJAZ11

MERDFLAAIGKEHPHPHKEKAAGAAEDSAYFGGARAGAAAAAAPAMDWSFASKPGAAPALMSFRSAAREEPSFPQFSSS
FDGAKNPAASRILTHQRSFGPDSTQYAAVHRAQQPPPQHALNGARVIPVSSPFNHNNNPMFRVQSSPSLPNGTAFKQPPFAI
NNAVPSSTVGFYGTRDAVRPKTAQLTIFYAGSVNVFDNVSAEKAQELMFLASRGSLPTSAPVARKPEAPIFAPAKVAVPEVL
PAKQMLFQRPQHVSSPPSAISKPIPGILQAATLPRSASSSNLDSPGPKSSVPLAVPPVSQAPAAQPATLATTTAAAIMPRAVPQ
ARKASLARFLEKRKERVTTAAPYPSAKSPLESSDTYGSGSANDKSSCTDIALSSNHEESLCLGQPRNISFSQESPSTKLQM

>SbJAZ12

MAGRAPATARDKTSFAATCSLLSQYLKEKKDGGLQRLGGLAMAPAAGAGAGGFRPPTTMNLLSALDAPAEEPTSDAAKA
TVEEPKDHHKSTAGNPREAAGDEAQQLTIFYGGKVVVFDKFPSTKVKDLLQIMNPGGDRVDRAGATATVPTQSLPTPSHN
SLSDLPIARRNSLHRFLEKRKDRITAKAPYQVNSSAGVEASFKVEKPWLGLGQEAATVKQEM

>SbJAZ13

MAASARPGERATSFTVACSLLSRFVRQNGAAAAELGLGIKGEVEQQRTPATISLLPGAEGEEAERTKETMELFPQSAGFGV
KDAAAPSEQENKEKPKQLTIFYGGKVLVFDDFPADKAKDLMQLASKGSLVVQNVVLPQPSAPAAVTDKAVPAPVISLSAA
QADAKKPARTNASDMPIMRKASLHRFLEKRKDRLNAKTPYQTSPSDAAPVKKEPESQAWLGLGPNAVKSNLSLS

>SbJAZ6

MAMAAAEGKSRRFALACGVLSQYVKAEQQMAAAAAPAARAPATTLSLMPGADVGAEQEPTPAAAARGEEMAGPASTA
APLTIFYGGRVVVFEDFPAEKAAEVMRLAAGGAERAAVPAPAPAPAPAPRDDLPIARKASLQRFLAKRKDRLVERAPYTRP
SPSPAEEAEKTKQPAASSWLGLGSTTDAERLTIAL

>SbJAZ7

MAPAKSGEKATSFAMACSLLSRYVRQNGAAAGDLGLAIRAEADGKRASADTEKGDTTKETMDLFPQNAGFGSEAAMKE
APDVREPEKRQLTIFYGGKVLVFNDFPAEKAKDLMQMASKGSSVAQNPGMLPSPTVATVTDSTKITAVPAAPIAVVNAQKS
PADIPQAPKASLRRFLEKRKDRLTAKAPYQGSPSDATPVKKEMAEGQPWLGLGPQIANPDLSLCKEGSQ

>SbJAZ8

MAAEQQQQPAKAAAAGSRFAVTCGLLRQYMKEQGGSGATRCLAPAVAMGLMPEADAAAAATEERTTVLELFPQQAGTL
KDEQQRKRKEPADGRAPLTIFYGGKMVVFDDFPAEKAEELMQLAGSGGNAAPAAENALGQPSLTDMPLARKVSLKRFLE
KRKNRLTAADPYPAAASESSTKQPPAVKEEGAPWLGVSSALSLS

>SbJAZ4

MAPSPMAQAGCSGRRRFAVACSVLSLCVRAETAAAAAAAHSRSVAQAQAGAASPMLLMPGADVVSDETVPTPAPAQLTI
VYGGRVQAFSDVSADRAAEVMRVAARQDMPPAGGLADLQVARKASLKRVMGKRRDRLGARAPYAPARPPPPKEQQEK
DADIWLGLGISG

>SbJAZ2

MAASGSRNNRFGITCARLRQFMMEQNHRQLRMGDLVVGSSSFQRPLQLTPVPVATGPAGSWDTGATTLSLFPAGTGTEVI
RPEETKATLTIFYKGQVATFHNFPADRAKDLLQMAGSVTGKAPEKGFLQMADSVTGKAPEKGVMMTAVPGKAETSDEPA
DAGAGMPPIARKLTLQRFLRKRKNRIAGTDDDGDHNEDALPWKKRDSAGTGNNPAEDAPDDASWLRL

>SbJAZ16

MERDFLGVIDRAAGKGGGDVVVVEDSRTELAEAAQWQFPAKSGSGAPAFMSFRTARGEEGSEEFSISGFRPAGDALDGIK
KQTSLPAVISQHQQQQRQFGLSSSQVTAQQYPAAAHGGQRLQGMDYSAAALHHLPGGSRLVQPLSVRHPAPFNQANLMV
RSQSFHNGTGVPSRNQQPFTMSNGFGGSTVGVYGARNPRNQTSTQLTIFYNGSVNVFDNVPVEKAKELMMLASRASIPSP
PIASHKLDSPISAPAKVNVPEVFPARQIVIQKPEPCVPHVTSTSSPIPIVPQVVTLSRSTSHCTTEACGSKPAVQSPVIAPITQAT
SSQPLATTSAAAVTPRAVPQARKASLARFLEKRKERVTSVEPYPTSKSPLEGSDTVGNASAPTKSSFTDIAPASNYGTEPVR
LGQPRNISFSSEVCPSTKLQI

>SbJAZ14

MDTSSGDLTNGGGATSSAQTTTKPLTMFYNGGVAVFHLPQDKAEVVMNMAAGEDGGGGGRHLQPNHGDELLAKLRQG
MPAASKISLQRFFQKRKERLYGP

>SbJAZ10

MEGGRDDDGGGDAYRAAAAAKERRRRWCGDDEESSDGSSGGGGGGGVELSLRLRTGADDDGAASAATPPPAPVAQAA
EARRNMTIFYNGRVCAVDVTEVQARAIISMASEETLQAADHRRQQLMRGDGDDCRRRRLQDGDGDRSSSSTSAVPPRCA
RDSGLVRPAAAAAPSRLLAAAGVVGSSRQGGGGVAAPVVEIDPAAAAGLSMKRSLQLFLQKRKARTAAAVAPPYAAGGR
QAQAVRR

>SbJAZ15

MPSMAPVELDFLGLRPPAAAADHHHGGTTTTTTCSSIRGMKTSAIASIGAHQLRRVIVGDEAPNNKQKQQAPMTVFYNG
AVATFDGVSQDKAEAIMKMAMEVTTSNGGRVVRGDAFAGNLTKDMPLTRTKSLQQFLQKRKERLSGLGPYQLGIMGSG
GTSRVGAATTNKSFHVKEEAA

>PtJAZ11 *Populus trichocarpa*

MRGSGMQWSFSNKVSAIPQFLSFKSSLEDKPRKAVHDPVASSSGLMSISTADAFDSNQKTYSGLVQKNMAIDKQAGNHY
PVTTYGTQQFDAYSVNRPQDTRMFSISGQQNQTITVSMSSPILQSHFPPTGHNMLSNSIVPKPLGGVPVITPTSALPTLSSIIG
TTDLRDGTKSSGAPAQLTIFYAGSVCVYDDVSPEKAQAIMFLAGNGGSSGTPNKPISTPQAQAPIRRPPVSDIFAGNKSNTT
APISCIPSPISVTSSNTNDLATVKPVVSLASSVKQTEPPKPLNSPGPTSATLVPAVAVPQARKASLARFLEKRKERVTQTSPYN
VSKRSPESGSHGCDGASLSMNFSSSYPLPASN

>PtJAZ12

MERDFLGLGSKNNPVTIKEEATDTPLKDSVPMRGSGMQWSFSNKVSAIPQFLSFKSSMEDKPRKAVHDPMASSSSGYMSI
STADAFDSNQKSYSALIQKNMALDKQAGNHYAMTTYGKQHFDAYFANRPQDMRMFPISSQQNQTINVSMSSPILQSLFPP
TGHSMITSNSIVSKPLGGVPVITPASALPTPSSVIGTTDLRDVAKSSGAPAQLTIFYAGSVSVYDDVSPEKAQAIMLLAGNGG
SSGTQNKPISTPQAQAQAPIPGPPVGDIFVGNKINTTAPCSGMPSPISVTSSSTNDLAIVKPVVNLAPSVKHIEPTKPASSVGP
TSATLVPAVAVPQARKASLARFLEKRKERVMQTSPYNGSKKSPEGGAHRFDGMSLSMSTSSSFPLPASN

>PtJAZ13

MEAQQPDSRKEMKPREDMEVKKEQEVMGSCKEGAGLLEKNGVHHLWAPSYWPMTMATSRPNVTIPTPDQLTIFYGGSV
VVFDSIPAEKVHEIMLIAAAAVKPGDMKKSGSPTGTPVLTRSPSMQSTAAPQGQTYSRQNSICRMQAELPIARRQSLQRFF
KKRRDRLVSKSPYPTSPAGKEADTTEPGISAAPPPDAGCFGKPLASEELQPKVAANVV

>PtJAZ2

MANLAQNSCKSPDQISSFAKKCNLLSQYLKEKGSFGDISLGINGKAPEVKGPETSDLPATTLNLLTNMENSSEHITFRQKPV
ASSNMMKYMDFFPQFVGFSPSNYTEDAINKADNHLRKSSTMDPGPTQMTIFYAGKLTVFNDIPAGKAEEIMALATKGSSIS
PNGFPSDPSIIKVNSANSVAALDSNNAQQRLHLQSEAPNGSDVPHATRASLHRFFSKRKERVTARAPYQTHNPTHDLPSSS
RPKEDSNSLLALNEGQSSKQLELKL

>PtJAZ6

MSGSTEFVENMGKMCEKPSFSQTCSLLSQYLKERGSFGDLNLGMASNSESTPNKNGPSEMLRRSPSTMNLFPVSEKPGHI
SCQNMGAPRNFTSMDLFPQQAGFAPKEDVPMKLDSRCVKSATAEPQTAQMTIFYAGRVIVFNDFPADKAKEVMLLASKG
SSQIQNAFPSIPANSHPALAPNISKTPIESTISIPSSSNALPNFGNNLIQESMQPAPQPIANDLPIARRASLHRFLEKRKDRIIAK
APYQINPAATTSKPAESEFSWLGLAAPSTTH

>PtJAZ3

MANMAQKSGKPQDQISNFAQKCNLLSQYLKERGSFGDISLGINGKAEIKGLETPSSPATTLNLLNNMEISSDQITSRQNAM
ASANMMKFMDFFPQFVGSGPPDSTDDAINKADHLRKSSPMDPETAQMTIFYAGKVSVFNDFPADKAKEIMALAAKGSSIS
TDGCPSSAPAIRKVSSTNSVAALDSNKGQERLQLQSQANASDVPHARRASLHRFFSKRKDRVAARAPYQINNPTPDHPRPP
RSEEDSNPFLALDEGQSSEQLELKL

>PtJAZ4

MKMSRGTVELDFFGMSKENRSSSSKSKCFNRQRSFRDIQSAISKINPELLKSVIASGSASNKATPANGNQLSNKAFSVPSTP
KQDLPPFPALPVYFPLPRLNLENPPETAPLTIFYNGTVAVFDVPRDKAENILKLAEKGFSKTVVESVADPRTDHQQKLLESL
DGDLPIARRKSLQRFLEKRKERLTSATPYACTPKTRF

>PtJAZ7

MTFVASDLVPMGGQSNSCKEMKPQEDIEVKKEQEVTNGCGENLVSCKEGADLLEKKGVHPLWTPSSRPTIMATSGPNATI
PTPDQLTIFYGGSVVVFDAIPAEKVQEIMLIAAAAAAAVKPVDMKKSGSPDGTPVLTRSPSMQSTAAPHAQAYSRQNSFCR
MQAELPIARRHSLQRFFEKRRDRLVSKSPYPTSPEGKEADTTKPGISAAPSPDAGCFGKSLASDELQPKMSSAVVVKIFWL
LLDALDSMYNFSLKLVGFCSHVCPLQQKALVMSSEPAPFGRLLVLL

>PtJAZ9

MERDFLGLSSKKPSAVVKEEISSDGCKDIGFTKGSGMHWPFSNKVSTLHNLMSFKAAQEDKTKTIESDALVSSGFMSVLS
ADACDPGQKRSAAEIQMFPVANHAISISTGNPFFNNHYPATGQNMFGTTMKPQLLGGFPVTAPHSILPMVGPVAGVTDSS
VKAYGSPAQLTIFYAGAVNVYDDISPEKAQAIMFLAGNGSSISSKSAQPKVQVQAFSSKPAAADVSPVNQPIMSTPPCSSLS
SPSHTGAQSGSGSTSTEEIMATKTTGPVTIPVIKPDHPKTGNVVGSVATTTMIPSVPQARKASLARFLEKRKESDECRAIQP
QQEISRFCQPRTLLINSFATSAAGTSSLLARRRTTYNESQSRRELLRFCAAS

>PtJAZ8

MHHPHDVKMFPVSNHAIPISMGNHFFKNHYPATGQNMAGTTTKPQLLGGIPVTAPHSILPMVGSVAGVTDSSVRASGSPA
QLTIFYAGSVNVYDDISPEKAQAIMFLAGNGPSISSNLAQPIVQVQASSSKPAAADLSPVNQPIMSTPPCSRLSSPSHTGAQS
GSGSTSTEEIMATKTTGALTTHVTKPEHTKTANVVGSVTTTTMIPSVPQARKASLARFLEKRKERVMNAAPYNLNKKSPH
FTNPEPY

>PtJAZ5

MPNSNGQAAAAVPVQMNHIEAQLVDEEDGDNNNIAGGGEESIDNTNSIQFEDGGCSGVVGEAVAASDMYVGTNGGGGA
DYGLVTANNDQLTLSFQGEVYVFDAVAPDKVQAVLLLLGGYEIPSGIPAMGTAPINQRTPNHGIYDLSGTGRSIQPHRAASL
SRFREKRKERCFDKKIRYTVRKEVALRYYQHAFLIPCLIGETRLVLMEIIT

>PtJAZ10

MQPGETVSRSALEKPLHQLTEDDISQLTREDCRRYLKEKGMRRPSWNKSQAIQQVISLKTLLETTPETESPRRRLYIPPPDN
PPRAPANSSVSVGGESADAPILVSAEELVPSRQPDPPNPVVPADPPPPVFVAATENDSVSPRTTGAAKESAGQMTIFYCGKV
NVYDNVPRDKAQVIMHLAASPFAPPQEASSNVIPALWPIPCQLETPGVKATPNSTVVIFPNLPTVKGADDGQLPQEESNIA
REDNLEGSTSRKASLQRYLEKKKDRLKNKRKVAMTSASVDIYLNHRVGDQISNDHWNLNDACSSPQPRPPQTPNRCNSI
DNLAKNGSLSADLNEKDAPEI

>AtJAZ1 ***Arabidopsis thaliana***

MSSSMECSEFVGSRRFTGKKPSFSQTCSRLSQYLKENGSFGDLSLGMACKPDVNGTLGNSRQPTTTMSLFPCEASNMDS
MVQDVKPTNLFPRQPSFSSSSSSLPKEDVLKMTQTTRSVKPESQTAPLTIFYAGQVIVFNDFSAEKAKEVINLASKGTANSL
AKNQTDIRSNIATIANQVPHPRKTTTQEPIQSSPTPLTELPIARRASLHRFLEKRKDRVTSKAPYQLCDPAKASSNPQTTGN
MSWLGLAAEI

>AtJAZ2

MSSFSAECWDFSGRKPSFSQTCTRLSRYLKEKGSFGDLSLGMTCKPDVNGGSRQPTMMNLFPCEASGMDSSAGQEDIKP
KTMFPRQSSFSSSSSSGTKEDVQMIKETTKSVKPESQSAPLTIFYGGRVMVFDDFSAEKAKEVIDLANKGSAKSFTCFTAEV
NNNHSAYSQKEIASSPNPVCSPAKTAAQEPIQPNPASLACELPIARRASLHRFLEKRKDRITSKAPYQIDGSAEASSKPTNPA
WLSSR

>AtJAZ3

MERDFLGLGSKNSPITVKEETSESSRDSAPNRGMNWSFSNKVSASSSQFLSFRPTQEDRHRKSGNYHLPHSGSFMPSSVAD
VYDSTRKAPYSSVQGVRMFPNSNQHEETNAVSMSMPGFQSHHYAPGGRSFMNNNNNSQPLVGVPIMAPPISILPPPGSIVG
TTDIRSSSKPIGSPAQLTIFYAGSVCVYDDISPEKAKAIMLLAGNGSSMPQVFSPPQTHQQVVHHTRASVDSSAMPPSFMPT
ISYLSPEAGSSTNGLGATKATRGLTSTYHNNQANGSNINCPVPVSCSTNVMAPTVALPLARKASLARFLEKRKERVTSVSP
YCLDKKSSTDCRRSMSECISSSLSSAT

>AtJAZ4

MERDFLGLGSKLSPITVKEETNEDSAPSRGMMDWSFSSKVGSGPQFLSFGTSQQETRVNTVNDHLLSSAAMDQNQRTYF
SSLQEDRVFPGSSQQDQTTITVSMSEPNYINSFINHQHLGGSPIMAPPVSVFPAPTTIRSSSKPLPPQLTIFYAGSVLVYQDIAP

EKAQAIMLLAGNGPHAKPVSQPKPQKLVHHSLPTTDPPTMPPSFLPSISYIVSETRSSGSNGVTGLGPTKTKASLASTRNNQ
TAAFSMAPTVGLPQTRKASLARFLEKRKERVINVSPYYVDNKSSIDCRTLMSECVSCPPAHHLH

>AtJAZ5

MSSSNENAKAQAPEKSDFTRRCSLLSRYLKEKGSFGNIDLGLYRKPDSSLALPGKFDPPGKQNAMHKAGHSKGEPSTSSG
GKVKDVADLSESQPGSSQLTIFFGGKVLVYNEFPVDKAKEIMEVAKQAKPVTEINIQTPINDENNNNKSSMVLPDLNEPTD
NNHLTKEQQQQQEQNQIVERIARRASLHRFFAKRKDRAVARAPYQVNQNAGHHRYPPKPEIVTGQPLEAGQSSQRPPDN
AIGQTMAHIKSDGDKDDIMKIEEGQSSKDLDLRL

>AtJAZ6

MSTGQAPEKSNFSQRCSLLSRYLKEKGSFGNINMGLARKSDLELAGKFDLKGQQNVIKKVETSETRPFKLIQKFSIGEAST
STEDKAIYIDLSEPAKVAPESGNSQLTIFFGGKVMVFNEFPEDKAKEIMEVAKEANHVAVDSKNSQSHMNLDKSNVVIPDL
NEPTSSGNNEDQETGQQHQVVERIARRASLHRFFAKRKDRAVARAPYQVNQHGSHLPPKPEMVAPSIKSGQSSQHIATPPK
PKAHNHMPMEVDKKEGQSSKNLELKL

>AtJAZ7

MIIIIKNCDKPLLNFKEMEMQTKCDLELRLLTSSYDSDFHSSLDESSSSEISQPKQESQILTIFYNGHMCVSSDLTHLEANAIL
SLASRDVEEKSLSLRSSDGSDPPTIPNNSTRFHYQKASMKRSLHSFLQKRSLRIQATSPYHRYR

>AtJAZ8

MKLQQNCDLELRLFPTSYDSDSSDTTSVVESTSSGNPQPNEESQRITIFYNGKMCFSSDVTHLQARSIISIASREMKTKSSSN
GSDPPNKSTSFHHNQLPNPKASMKKSLQSFLQKRKIRIQATSPYHSRR

>AtJAZ9

MERDFLGLSDKQYLSNNVKHEVNDDAVEERGLSTKAAREWGKSKVFATSSFMPSSDFQEAKAFPGAYQWGSVSAANVF
RRCQFGGAFQNATPLLLGGSVPLPTHPSLVPRVASSGSSPQLTIFYGGTISVFNDISPDKAQAIMLCAGNGLKGETGDSKPV
REAERMYGKQIHNTAATSSSSATHTDNFSRCRDTPVAATNAMSMIESFNAAPRNMIPSVPQARKASLARFLEKRKERLMS
AMPYKKMLLDLSTGESSGMNYSSTSPT

>AtJAZ10

MSKATIELDFLGLEKKQTNNAPKPKFQKFLDRRRSFRDIQGAISKIDPEIIKSLLASTGNNSDSSAKSRSVPSTPREDQPQIPI
SPVHASLARSSTELVSGTVPMTIFYNGSVSVFQVSRNKAGEIMKVANEAASKKDESSMETDLSVILPTTLRPKLFGQNLEG
DLPIARRKSLQRFLEKRKERLVSTSPYYPTSA

>AtJAZ11

MAEVNGDFPVPSFADGTGSVSAGLDLLVERSIHEARSTEPDASTQLTIIFGGSCRVFNGVPAQKVQEIIRIAFAGKQTKNVT
GINPALNRALSFSTVADLPIARRRSLQRFLEKRRDRSTKPDGSMILPSQLTIIFGGSFSVFDGIPAEKVQEILHIAAAAKATETI
NLTSINPALKRAISFSNASTVACVSTADVPIARRRSLQRFFEKRRHRFVHTKPYSATTSEADKNETSPIVT

>AtJAZ12

MTKVKDEPRASVEGGCGVADGDGGAAEIGGTGSVEKSINEVRSTEIQTAEPTVPPNQLTIFFGGSVTVFDGLPSEKVQEILR
IAAKAMETKNSTSISPVSSPALNRAPSFSSTSNVASPAAQPFPIQPISFCRSTADLPIARRHSLQRFLEKRRDRLVNKNPYPTS
DFKKTDVPTGNVSIKEEFPTA

>AtJAZ13

MKGCSLDLHLSPMASTLQSCHQDSTVNDRSSTIRSKEINAFYSGRLSEYDLVEIQMRAIIEMASKDREVTALELVPVRLESP
LGCSVKRSVKRFLEKRKKRSKSFTLTPNYTSSTSSSSSSLHNF

>OsJAZ1 *Oryza sativa*

MASTDPMTRRFAVACGVLSQYVKANSSQPSTAAPVAQGVSGLMAAAAAAAAAPVVQEPGCEVDGGGQQFTIFYAGKVV
VIDRCTPAMAAELMRFASAAQGGGGAPEAPPALVDMPIARKASLKRFLAKRKATPASARSSYVVRAAAAEEEQPPAKKA
KAAVERREDWLALGSLGHMHSR

>OsJAZ2

MAGSSEQQLVANAAATTVAGNGSRFAVTCGLLRQYMKEHSGSNGGGGFLPAVTAMSLMTGGADAEEEAPEVRKTMELF

PQQAGTLKDTQERKEITEKAQLTIFYGGSVVVFDDFPAEKAGELMKLAGSRDSTAAAAVSDAGAAAGQPCLPDMPIARK
VSLQRFLEKRKNRIVVAEPLPESEKKEAESSKRAKKDDGGASWLQVNPTLSL

>OsJAZ3

MAMEGKSRRFAVACGVLSQYVRAEQKMAAAAGAAPARAVTTLSLMPGAEVVVEEEERREVGEEEAGPATAPAAPLTIFY
GGRMVVFEDFPADKAAEVMRMASSGMAAAPAQREGAALADMPIMRKASLQRFFAKRKDRLAATTPYARPSPAETKASE
PEEKKTPTSWLDLAASASAAARRDSLTIAL

>OsJAZ4

MDAVGAAGGGAMLPAAARRGQPPQPPCMTTAPEQQAAAGGAVIWPAAAAAEAKEKMVVDA
RTMQLFPTRSADGVVVSPAPAPAAAQERRRPEVHVTPSVPATAPTAPLTIVYGGQVLVFEHYTAEAAEKLVQRTQHLLAAA
AGGGGGNKNNNVTVVTPPPDEPPMLLPPPQMPAASGVSAGGVMPIARKASLQRFLQKRKQK

>OsJAZ5

MASAKSGERGSSSFAMACSLLSRYVRQNGAAAGELGLGIRGEADANKGKETMELFPQNSGFGSEAAAVKETPDAREQEK
RQLTIFYGGKVLVFDDFPAEKAKDLMQMASKSSSTAQNCVLLPSSATATVADNTKVSAVPAPASALPVAQANAPKPVRPN
AADLPQARKASLHRFLEKRKDRLQAKAPYQGSPSDASPVKKELQESQPWLGLGPQVAAPDLSLRQESSQ

>OsJAZ6

MSTRAPVELDFLGLRAAAADADDRHAKSGGSSASSSSSIRGMETSAIARIGPHLLRRVIAAAGPPPPPSTAPVPEEMPGAA
AAAAPMTLFYNGSVAVFDVSHDKAEAIMRMATEATKAKGLARGNAIVGNFAKEPLTRTKSLQRFLSKRKERLTSLGPYQV
GGPAAVGATTSTTTKSFLAKEEEHTAS

>OsJAZ7

MDLLEKKNIKKGGEVEEEVARKGEERKEEEVVVEEKSHQQQQQQGEEELVGLSLAGGRPKVFPMSSPPPNPSQLTIFYGG
SVCVYDSVPPEKAQAIMLIAAAAAAAASATKSNAAIAVKPPVMPAANATQAAVSPVLTRSLSLQSTSVATGQPQVAADPSS
ICKLQADLPIARRHSLQRFLEKRRDRLVSKAPYPTKSSEGMEASGMEVTAEGKAQ

>OsJAZ8

MAEERRRDDGGDVEVELSLRLRTGDDSTSADPAPATVAAEARRNLTIFYNGRMCAVNVTELQARTIISMASQGNFGKQQQ
QQIQGRDDHHYHQGESSSGGGVSTAAARHCDVAGSSSSHSGSGSGSATPPRPALVSPRAGLQAAAAAAPTMNQPPAASGL
SMKRSLQRFLEKRKTRAAAPLYARR

>OsJAZ9

MAASARPVGVGGERATSFAMACSLLSRYVRQNGAAAAELGLGIRGEGEAPRAAPATMSLLPGEAERKKETMELFPQSAG
FGQQDAITADSAADAREQEPEKRQLTIFYGGKVLVFNDFPADKAKGLMQLASKGSPVAPQNAAAPAPAAVTDNTKAPMA
VPAPVSSLPTAQADAQKPARANASDMPIARKASLHRFLEKRKDRLNAKTPYQASPSDATPVKKEPESQPWLGLGPNAVV
KPIERGQ

>OsJAZ10

MERDFLGAIWRKEEAAGKPEEHSVSRDADYRGGGGGASAAMQWQFPATKVGAASSAFMSFRSSAAAAREEDPKEAAVF
DRFSLSGFRPPPRPSPGDAFDGAAAMKQRQFGFNGRQQYAAAAQHGHREQGVDSYGVAAPHHFPSPSPSPRHPVPFGHA
NPMLRVHSLPNVAGGSPYRNQSFSVGNSVAGSTVGVYGGPRDLQNPKVTQMTIFYDGLVNVFDNIPVEKAQELMLLASR
ASIPSPPSAARKSDSPISAAAKLTVPEALPARQIVVQKPEASVPLVSGVSNPITIVSQAVTLPKSFSSSNDSAGPKSGGLPLAV
TPLSQASPSQPIPVATTNASAIMPRAVPQARKASLARFLEKRKERVSSVAPYPSSKSPLESSDTIGSPSTPSKSSCTDITPSTNN
CEDSLCLGQPRNISFSSQEPPSTKLQI

>OsJAZ11

MERDFLGAIGKDEEQRRHAEERKESDYFGAGGGAAAAAMDWSFASRAALMSFRSSSSAAAAAAREETRELAFPHFSAL
DGAKMQQASHVLARQKSFGAESHGIPQYAAAAAVHGAHRGQPPHVLNGARVIPASSPFNPNNPMFRVQSSPNLPNAVGA
GGGAFKQPPFAMGNAVAGSTVGVYGTRDMPKAKAAQLTIFYAGSVNVFNNVSPEKAQELMFLASRGSLPSAPTTVARMP
EAHVFPPAKVTVPEVSPTKPMMLQKPQLVSSPVPAISKPISVVSQATSLPRSASSSNVDSNVTKSSGPLVVPPTSLPPPAQPE
TLATTTAAAIMPRAVPQARKASLARFLEKRKERVTTVAPYPLAKSPLESSDTMGSANDNKSSCTDIALSSNRDESLSLGQP

RTISFCEESPSTKLQI

>OsJAZ12

MAGRATATATAAGKDRSSFAVTCSLLSQFLKEKKGGGGGLQGLGLGLRPAPAAPPAAGAGGAFRPPPTTMNLLSGLDAPA
VEVEPNTAETAADELPLIKAPADQQSDESASEAAGEKAQQLTIFYGGKVVVFENFPSTKVKDLLQIVSTGDGVDKNTGTA
ATQSLPRPAHNSLPDLPIARRNSLHRFLEKRKGRMNANAPYQANCTAAPSKQANGDKSWLGFGQEMTIKQEI

>OsJAZ13

MAAEAAATSRFAAACGALSQYVRAADNVHRARTAAAAAAVRPLPLMPGADVAGDEREEEGGGAAASSAAAQMTIFYG
GRVLVLDECPADRAAALLRLAASSRGVPRDDLASTAAAAGESADLPVARKASLQRFMEKRKGRLAARGQPYRRHDAAA
AARGDHLALAL

>OsJAZ14

MAVSDHHCGGGGRSWRFAVACGVLSRCVKAEAAAAANGRHRHHPTMLLMPGADVEPDVREEAAAAAQLKIMYGGRM
LVFDDFFPAGGAVVELVRAAARAGQDVRRAGAARRRVGDSRGLDAGLPVVRKVSLQRFVEKRRRMRVYHILYTDKSSH
HVPGPGRYRSWQCRIIIAAVAGAGGFVVACGVLSRCVKAEAAAAAANGRRHHHHHHTTMLLMPGADVEPDVREEAAA
AAQLKIMYGGRMLVFDDFFPAGGAVVELVRAAARAGRDDDGARARRRPAGGEEGVAAAVRGEEKSQAARGDGAVHTR
HSPPMLPARTPGSGRTDDAAFY

>OsJAZ15

MAAAGSSSRFAVTCGLLSQYMRERQQPQPPVTVLEAVAEEEEEEDARTMQLFPPRAAAADGVATPSAGTAPLTIFYDGRM
VVVDDVPVEKAAELMRLAGSACSPPQPAHAAALPEMPIARKASLQRFLQKRKHRITTTSEPYKKAAVASPAPEKSFAVAP
VKDEPATWLGL

>PpJAZ2

MSKMSVIPEQLSSRDYDDDNNNNFNNSSSSSSSSRRRDLDNSFYSRPGLSQSPWLNKHSMAKRVPADPARIEENSVTAVID
TSLTKETMEGSHPTMNSRNPYEDFALVQQAGTATSMHIDLLQSFKSNVRTAPHIGNAQLSAHRSDPSASEPSSLPQQHPSSN
SARFVGLAPFFSRMRGNSRVENESLPQLQHQQATAHAFSRPPGTGKLIESAQLSIFYAGMVNVYDAVPIEKAQAIMLLAGT
RSAWSSPNHMNLPGAPGHPFPASINQPPFRTRSPGPQMISGYAGTTALSGVPKATNRQVPTAETGFIVELPQARKASLARFL
DKRKDRVRKGPYNDPRNENLARYDELRTSRENSPCPSDSKGKAIALSLSPSATRNQRNSPFPGDPGHASSASASSSEPTSRE
TME

>PpJAZ1 *Physcomitrella patens*

MGIEGATQPQVRPIPSASGSHLAEAPAMSRMPPLVEGVCSRDSSNNNMDRDTSFFARPGVTRSPWMNMPTITKTLPADSA
RSEDNSVMTVDDVFLKKEVMQSRFEDSALSQQARKVASMNVNHLRQAASHVFARAPGSSKQPPTAQLTIFYAGMVNVF
DDVPLDKAQAIMLLAGTDSTCSSNHMNLPGASVRPFPTRMSQPSSRVGSPAPQMTTSSAGTAALPGAPRAVNRQALTAAT
TGLIVELPQARKASLARFLEKRKDRVRKGPYTDSRNEEAARDDESRRSGRQNSPCPSDPKGKIPARSLSPPATKNQGNAPF
PGGASTSFSELNSPPQTPPRKSMSDDRSHETLEKSKESESRIEAMVPSGEKLSSPSRNGTGSRTEPMDEHSS

>PpJAZ3

MARDAVAVDFMGIGERRGGVSYDDNEERVRLVRPVPTVPEDRQGQSRTWPGAEEWRNPGDRDASPFTQRLPALHRSPWS
RLPIASRFQSDEENSSKAMVSPKREKDLKEGLPTGHQQSFSAWDNSTPGNPSYHPTGSSSRYEEFVRMQQHLQARKAAV
MHADHQQAPRCVQNAGARTSPFRDEAQRGAQPSDRLGPEHLTTQPHAPSNQSGLGSPGPFHGRGSGTAFTGRRIPFQAG
NLSNHVVRRGGTGAKPRTAQLTIFYAGMVNVYDDVPFDKAQAIMLLAGSGSTWSSNNMGHRGSGPARPFSAPTAVPQPT
PSTPGSPAPQGSTTSAAGSLRPVIPGVMFSSVRQPPVANVELPQARKASLARFLEKRKDRVRKVPVKAEGETSPSRDKSPTP
SCGNAPSRSSSPCPVGQERGSSPACGQGHQPGASSCSEPNSPTIPSQTPPTESASEEKSSIGTPKRKDVEMEQESHKRARIGR
SPPRVAGRSTNDVMEQHES

>PpJAZ6

MTREPVAIDLINLGGIAERGVGGLMRSSSIECDERVSRGRLVRPVPSMLNHPSRSQSVSRMSPGPDGLRDRNINYFTQPGPF
RPPWMSNHVAAGRVLTDCRSHCNFGMAAETSAERNMVSEDHLTGSQSRGDHSAPASPSHHIRLSSSSGYELDFTQQHTR
KYASIFTSPAHALRNMPASTSSLREEAQAMSTHQSNLTAAEPSSVPRQHPSSSHSAGFGSPVPFYSRMKDNTGAESGSALG

SQNPPRQPELQHPVKDTKMAAAHGLVRSATVGELPRVAQLTIFYAGMVNVYDHVPYEKAQAIMLLAGRESYPNYESLLG
GCSATESPWICSPGAINLQASRGGSPAPLSSADLPPPGVVPMAIRPTPTTTAVELPQARKASLARFLERRRDRVRTGPYVPR
NEEARRIRENPPSPSVSSARPPTRPSSPVPGHNNTAPPNAGGATTSTSQVNAPPPTPSRESSSEVLDPSPGRPQGENGEGMQP
APPGVSSVGEGSVAMS

>PpJAZ7

MAGEPVAVDYMGVGGAPTSSPDGTEERVRLVRPIPTVPDNRAGNFRISPAPEGGRDNSPFVHHGLRQQAWNRVQVAARF
QLENENSNKVVVCPKKEKETTDGPLNGHPQFSSQWDNSAPTSPSHHLGSSANRYDAFLRMHQSEKVTTTYGNHQALKG
MQNGGGRTSPFRDEAQRGAQPSDHATQPHPPSRQGGCGAPTPFCARSNSGVVDSVAAMAGKKLPFQHPVQSGSLPRPGG
LGKQPRTAQLTIFYAGMVNVYDDVPFDKAQAITLLAGSRNTWSSNFMNPPQAGSAASGRTFSTPTAVPPSTPSTPGSPAPQ
ASTTSAPAPPRPSLSGVVFSNVRQPPIHNFELPQARKASLARFLEKRKDRVKKVIVKEEEDASPPRGNSEGPSGGKPPSRSP
SPSPAVSRSMDYLSSPGRVEHQAGTSSCSERSSPSNPRPPQTPPSQSQSEERSAGTTKRRGFGLAHEPNKRARNGKSQCRLS
GESAADHIVEDGSFPHR

>PpJAZ5

MVDWRAMAREPVAVDYMGVGGAPSSISGGHEERVSLVRPIPSVPNNGGGHFRRSPAPEGGPHSSPFEQHDPDQKSWKRA
SFLARFQSEEDNSRRVIICPNKERESTDGHLNDHPQSSSQWNNSESKSHTHHPQSQKVAAMYASHHHLLRGIQNGDAWTS
PLKDEVQRGAQPLDHAMQPHLSSNQGRFESPMPFGGHRLGSVESGAAVARKKPPFQYPFQAGNLPRPGGPCKQPRTAQL
TIFYAGMVNLYDDVPVDKAQAIMLFAGSESTWSSNLMDPPQAGSVASGRTFSAPTTVPLSTPGPPAPQALTTSASGPPSSVL
PGMVFSNLRQPSTTNVELPQARKASLARFLEKRKDRVKKDPVKEGDATPFGNSPDPSIGKPPTWSPSPSPSVSRCMDHGSS
PGRLEHQSGTSSGNEQNSPCNSRPPQSPPSQGVAE

>PpJAZ4

MSDAEPLVHPQSLQLHHSLHPHNLNHGLSHAQHAMHEMHVHGHGEADGHGHVRVDQRVQGHLEGDHAHGNHGHHG
HGMHNNEENEAEVEDHDDDAADEEGLDEANMHSDGGGNPNDGPAPLTVRTQSSTQLTLSYQGEVYVFDAVPPEKVQAV
LLLLGGREIPPGMSGVNISNNHHHHKGLTDLPARMNMPQRLASLTRFREKRKERCYDKKIRYTVRKEVAQRMQRKKGQF
ASSRPSQEEGAPVANWDGTQALGQPVGAGGVQPEVICVHCGIGERSTPMMRRGPAGPRTLCNACGLMWANKGVLRDLS
KNLSIAPGVQQQLILQSQQIILQQLQHQVAGSQHSAENTSQVVDVQGSLQGEVDVQKNIAGIVAAGGGPVLAAG

>SmJAZ1 *Selaginella moellendorffii*

MAAGHNETELVRTLKQIIELRGLKQQRRSSAATDTVTLFGKKLSVGGGGGSSLEESSTDKIQLSEKLPPSAIVHLKELYEQR
LKRKAAPCHSARSAENGDALTLPLLGSIQDVGIKRQRIGVQAAAATEDVRPIFYEIQSDLAAEKERAEVAKGAPLTLFYNG
MVYVFDDVTDDMAQAIMILAGNATCSSASHTEKFLASAAKDAKPAAAMPSFTLADLPQARKASLHRFLEKRKDRLFAKS
DKESVSSSKPKTPPRSPPRKQHHLLREMIQPPSFLQIY

>SmJAZ3

MVLEARELQFRKLLTGDDGFNDSATLSLLPPSQVSSSQLEVPPERPLTHAAPLVNGARPKHQLTIFYAGCVNVYDDIPEDK
AHAIMLLAGNSATGNASQILGSLASLQAPAANHAGFPEPAHKVRKLQQGLAAPSQAGLLLSLNFGGRASGGNFNPGIPKIS
CPPVPAQVLPQARKASLTRFLQRRKERVLQQSMESVKDDKTSSEFSVKNQDCASAQEENSALKKENSVGSIEA

>SmJAZ4

MSRARTDSKLFRSTGSVGKPGRKPAVGGSPVAAAAASSIPVKESPDSSQQVDPDLTRALKQIFEVRNCKLKDHGSSHNNN
SNSSSKNSSEESCLTLFGRKLASPSSSLDGSDGNTKNVANQYSRQGTDKLPPAATAHLLKELHKQRLKQRGITASANDFPD
MGEIEFVHTERRPEVSTLPLLNNLQEKRPLENASDCSPEAKRQRIEKDKSLDLSLSVEFGSPDEAARDSNNTQSMDIAAEE
ERTHATVKDPEQLTLFYNGQVLVYDDVPSDKAKAIMVLASSISSKTSCYTPCLAAATAAAAKATSAATAPPIVVPTTAKAS
NSLYPSPKGTTPAADKRSSSNDVQARKASISRFLVKRKDRLSAKPAEKAEASSKSSAKDISQRSRSPSPAASSSPVTPPRASP
PRLVDELKLQLEKGEMLKRSIPANAGSAAAAAAAPRQWIVFFSSGEHIRLMDGWMDGRLITNNALGKIVEGCFV

>SmJAZ6

MVSVRPGRIEQHKQVSPDRMMFKVPANSPPPPPSSSSQAPADMAGKRKWQEAFKGESCPSVPAPPHRSPQLTIFYAGAVN
VYDNVPENMVKALMTLAAAKSSRPAALMNQIELQRSCSSNKTGFGFQANQSSCNGGGSARAGVKSSSNVASSSTSGSFS

FPVLETTSTSLSLNSSYKAANEERSSVPAPAPAAAAAPAPEQSSGTAVQASSSPPSPAASPDLRGMSSRARMQMLPAGLPQA

RRASIQRFLQKRKQKGTVIVNRQNNNGEDDDEDAATAEMKPTSPPPVGPPTS

ASTAHIAAQVPHFLKKR

>SmJAZ5

MESDSISGFTDERYLRDPLSCLGTEALIDLFSPPRYNVASSLDYSQDFEGDRGQSHSSNKNLLEIPEEQIDDEEIEDDRECLR

RDFTNPGNSEEEEEKEKETSDSGLQKEDTYSQATGAKNPDSSRASLEAIDDAKALKTLDEHRPDVVGVVETQAKDCSHGI

MVVNEAELEEVVDTRGKNDKSDLVTGMMRRGEKNHDSNDSHATRIDPSPEEDSLKGGGWSLPYMGQPGSVISSKPHRV

SGRCKSSRAMADAAVLASSVITSFIQGVKALKDASVNLDEAPSNFQNLKRLVTQLESSVTKTEKKYANKLHNDEFRARLR

DLRELVQEFSTEVGKAERLTSRKGLTRVMACCWSAAVGDELLRIAYSMESQLQKWFEQQNVPVSIEEAMERYAQGLPSYL

TLQPEDGYQPLRSKVQEVCDLLASSPHKVVLVHGLSGIGKSSLAHFVGASSLPTRFVDGSLKVLLGYGCSRAALGNNTKE

YQKDFAEKIVHLLRTQLGYKKHDLDGISLKEAFVILEETLKEKNYLIIVDDVWEADVVIRFMKLQGNRCKYLVTTRLYAM

VSSDVDRVEVTKEDVAQVGKEILRHHSQVQDLPELADELLHRCGHHPLTVTVIGQALEGESRHEQWLQAINDLSIYASRA

PVPNKDLMDDDVSNAATVFGSLEFSLKAMEKETREFFTAFAALSWVEPIPEPCLEEMWRALGLQGTFRLAIGALLKGSLI

GKHKSSYSYIIHDMVALYLAEKDLESLELLKLPDSSRELATVAPWLYRYGKERPRKMAGDALLRILGSRNEALQASVFDAT

INLCSLCKTYQDWSETSQSFVLLLGPGIPRLIPELGPGGKAAQSALRFIANYYTPSDWEYNLSSFIDTGILHHIRQLLKPDDD

SLSSMVHVLKKLVTFISAAENAQDTLRIMPIDELVKLLDPSSKTTRNFVGSSAMDALIAMAEAGGQDVVDRMFSAGLGA

MMVQHIRSDFDEFGGCGMKELNVLRERYLEGGSTIVATFRRSKLYDKMPERHAAFFDDSFSLTCDVDGTGKAWIALITTI

VCCEFCERISMWYPQDIHPSTSAHICIAFELQSDPHHTSGYGCSSKESQSFMGEYSLRFPVEALAGEVFAMANGMGSTRDL

SGGAANGSRVSLQLDGDGEAASLSRLQQMSRQTGFQLAAAFGSASNSRNAEHALSSRQGFGFMQSYMTAVAGVCNPGR

WSLRPWSHYKTTENQGQSVLLKPTPNLSHNTDKTGDLSLSLRPPSSSSSPPTPKQPAFSGEFTMLYDGKVAVYESMPIDKA

QAIMLLAGSVTSGSSETAAAAVNLLLSANNISQSSSSNSKCSALPQARKASLQRFLEKRKER

>SmJAZ2

MASGMESTRDLSGGAANGSRVSLQLDGDGEAASLSRLQQMSRQTGFQLAAAFGSASSSRNAEHALSSRQGFGFMQSYM

TAGVCNPGRWSLRPWSHYKTTENQGQSVLLKLTPNLPHNTDKTGDLSLSLGPPSSSSSPPTPKQPAFSGEFTMLYDGKVAV

YESMPIDKAQAIMLLAGSVASGSSETAAAAVNLLLSANNISQSSSSNSKCSALPQARKASLQRFLEKRKESQIKEVISFSSSP

PDAHRLLLIQSKKPHLFLVVPTAILTFHARAVVVRPANVVVVDHRDEELGLGRGCKLRGRTAELRRRPAAASGGRLRRAA

VPGRGGDHRAPAGGVERIGAAAVEEDPGRVLEGAAFALWMLLTSGSAIKLDQASKMEILSNKAEVVDRMFSGDDGPAH

MVRLQRVWACGMNRLRERYLEGVETFRRSKLYDKTSERLFD
